# On-Surface Synthesis and Characterization of Pentadecacene and Its Gold Complexes

**DOI:** 10.1021/jacs.4c13296

**Published:** 2025-01-17

**Authors:** Zilin Ruan, Jakob Schramm, John B. Bauer, Tim Naumann, Laurentia V. Müller, Felix Sättele, Holger F. Bettinger, Ralf Tonner-Zech, J. Michael Gottfried

**Affiliations:** † Philipps-Universität Marburg, Fachbereich Chemie, Hans-Meerwein-Str. 4, 35032 Marburg, Germany; ‡ Universität Leipzig, Fakultät für Chemie und Mineralogie, Wilhelm-Ostwald-Institut für Physikalische und Theoretische Chemie, Linnéstraße 2, 04103 Leipzig, Germany; § Universität Tübingen, Institut für Organische Chemie, Auf der Morgenstelle 18, 72076 Tübingen, Germany

## Abstract

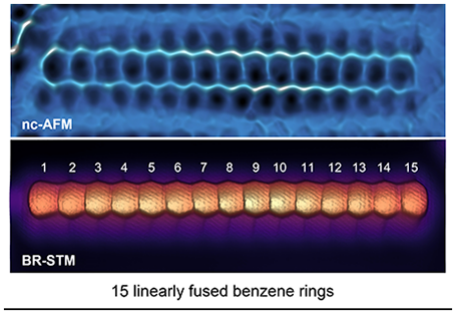

Acenes are an important class of polycyclic aromatic hydrocarbons that have gained considerable attention from chemists, physicists, and material scientists, due to their exceptional potential for organic electronics. They serve as an ideal platform for studying the physical and chemical properties of sp^2^ carbon frameworks in the one-dimensional limit and also provide a fertile playground to explore magnetism in graphenic nanostructures due to their zigzag edge topology. While higher acenes up to tridecacene have been successfully generated by means of on-surface synthesis, it is imperative to extend their synthesis toward even longer homologues to comprehensively understand the evolution of their electronic ground state. Here, we demonstrate the on-surface synthesis of pentadecacene (**15ac**) from a trietheno-bridged precursor via the atom-manipulation-induced dissociation of protecting groups or the elimination of adatoms in a gold-pentadecacene complex. The generated **15ac** was investigated by scanning tunneling microscopy (STM)/spectroscopy (STS) and noncontact atomic force microscopy (nc-AFM), in combination with first-principles spin-polarized density functional theory (DFT) calculations. We found that **15ac** exhibits an open-shell singlet ground state with an experimental singlet–triplet gap of 124 meV and an STS transport gap of ∼1.12 eV. The formation of Au-pentadecacene complexes suggested a considerable contribution of polyradical character to the electronic ground state of **15ac**. Our work contributes to a fundamental understanding of the electronic properties of long acenes and to the development of a versatile STM tip-assisted methodology for the synthesis of elusive compounds.

## Introduction

Acenes are an important fundamental class of organic compounds constituted by linearly fused benzene rings.^1^ A unique feature of acenes is the presence of only one Clar sextet, indicating that the aromatic stabilization is reduced as the size of the acenes increases.^2^ Consequently, long acenes have a relatively narrow gap between the highest occupied and lowest unoccupied molecular orbitals (HOMO–LUMO gap), which eventually results in their open-shell polyradical character,^3−5^ making acenes promising candidates for application in spintronics.^6^ However, the open-shell character of long acenes also leads to low kinetic stability, posing a significant challenge for the chemical synthesis of long acenes.^7^ To address this instability, a recurrent strategy involves the functionalization of long acenes with protecting groups.^8−11^ Unsubstituted acenes up to undecacene have been successfully synthesized by means of matrix isolation techniques.^12−16^ Additionally, unsubstituted acenes of different lengths, including the longest reported acenes, dodecacene and tridecacene, have been generated on metal surfaces under ultrahigh-vacuum (UHV) conditions by thermally driven or light-driven chemical reactions,^17−22^ as well as by atom manipulation at low temperature using the tip of a scanning probe microscope (SPM).^23,24^ This on-surface synthesis approach allows for subsequent structural and electronic characterization of generated acenes at the single-molecule level, which also enables direct comparison of the properties of different acenes.^17,25^

Up to date, tridecacene (**13ac**) is the longest acene that has been synthesized^22,24^ and experimental evidence for its open-shell electronic ground state has been reported.^22^ In the latter work, thermally induced dehydrogenation reaction of a hydroacene precursor on a Au(111) surface was used to generate **13ac**, which showed a HOMO–LUMO gap of 1.40 eV and a spin excitation feature around the Fermi level in scanning tunneling spectroscopy (STS). This feature was attributed to the transition from the singlet ground state to a triplet state.^22^ In a parallel work,^24^ in which **13ac** was generated by SPM tip manipulation at 4 K on Au(111), a smaller gap of 1.09 eV and no spin excitation feature were observed. It has been speculated^24^ that the deviating findings in these two studies may be related to the different methods of preparation, which may result e.g. in different adsorption sites of the **13ac** and thus different molecule–surface interactions. To resolve these discrepancies and to unambiguously unveil the open-shell polyradical character for long acenes as predicted by theoretical investigations,^7,26^ it is thus imperative to synthesize and characterize acenes longer than **13ac**.

**Scheme 1:**
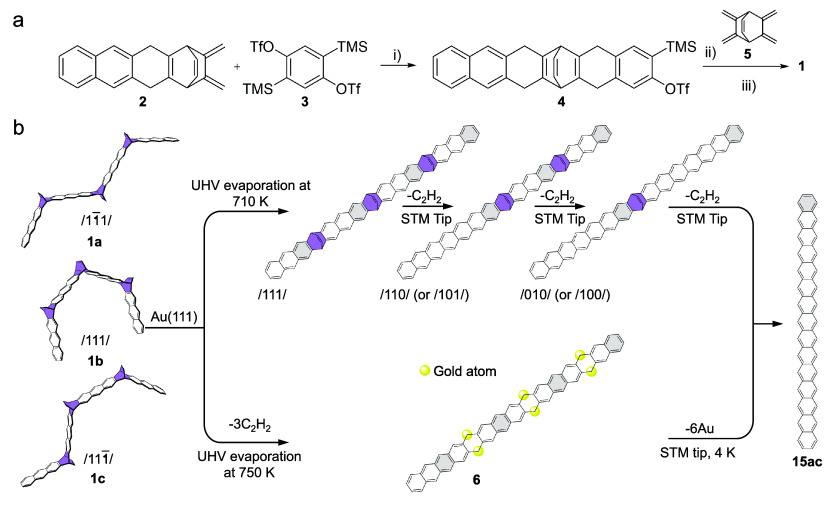
Combined In-Solution and On-Surface Generation of Pentadecacene (**15ac**) (a) In-solution synthesis of the precursor molecule **1** (three stereoisomers **1a**-**1c**). Reagents and conditions: (i) MeCN, CsF, 45 °C, 19 h, 37%; (ii) DCM, MeCN, KF, 18-crown-6, RT, 16 h, 26%; (iii) DDQ, CHCl_3_, RT, 3.5 h, 8%. (b) On-surface synthesis of **15ac**. The stereoisomers (**1a**-**1c**), in which the etheno-bridges can be on the same side of the backbone (/111/) or on different sides (/11̅1/and/111̅/) are used as the molecular precursor. Upper path: Sublimation of the mixture of **1** at 710 K onto Au(111) leads to the formation of the stretched-out/111/isomer. Sequential removal of the etheno-bridges using manipulation with the STM tip, producing **15ac** via the dietheno-bridged (/110/ and /101/) and monoetheno-bridged intermediates. Lower path: High-temperature sublimation of the mixture of **1** at 750 K induces the cleavage of the trietheno-bridges due to solid-state pyrolysis and the generated **15ac** spontaneously forms a metal complex with up to 6 gold atoms (**6**). Tip-assisted removal of the Au atoms generates **15ac**.

In this work, we report the generation of pentadecacene (**15ac**, Scheme 1), the longest acene generated to date, by a combined in-solution and on-surface approach, and the detailed investigation of its ground state electronic structure by scanning tunneling microscopy/spectroscopy (STM/S), noncontact atomic force microscopy (nc-AFM), and (spin-polarized) density functional theory (DFT) calculations. The on-surface generation of **15ac** is achieved by a multistep STM tip manipulation of a hexahydro-trietheno-pentadecacene precursor on Au(111). The tip assisted removal of etheno bridges (or gold adatoms) produces intermediates with fewer Clar sextets than the precursor due to extension of the π-electron system. Our experimental investigations of **15ac** reveal an STS transport gap of around 1.12 eV, which is comparable to that of **13ac** (1.09 eV).^24^ Low-bias differential conductance spectroscopy (dI/dV) shows a singlet–triplet spin excitation feature, indicating a singlet–triplet gap of 124 mV, which is comparable to that of **13ac** (126 meV).^22^ Theoretical calculations show that the interaction with the surface leads to a lowering of the magnetization of **15ac**. As a result, the tetraradical character present in the gas phase is reduced on the surface. Additionally, theory predicts that the energy gap between the highest occupied molecular orbital (HOMO) and the lowest unoccupied molecular orbital (LUMO) is reduced upon adsorption, which is similar to the findings for **13ac**.^24^ Moreover, we show that **15ac** forms metal complexes with up to six gold atoms when deposited to the Au(111) surface held at 150 K (Scheme 1, compound **6**), in line with considerable polyradical contributions to its electronic ground state.

## Results and Discussion

**Figure 1:**
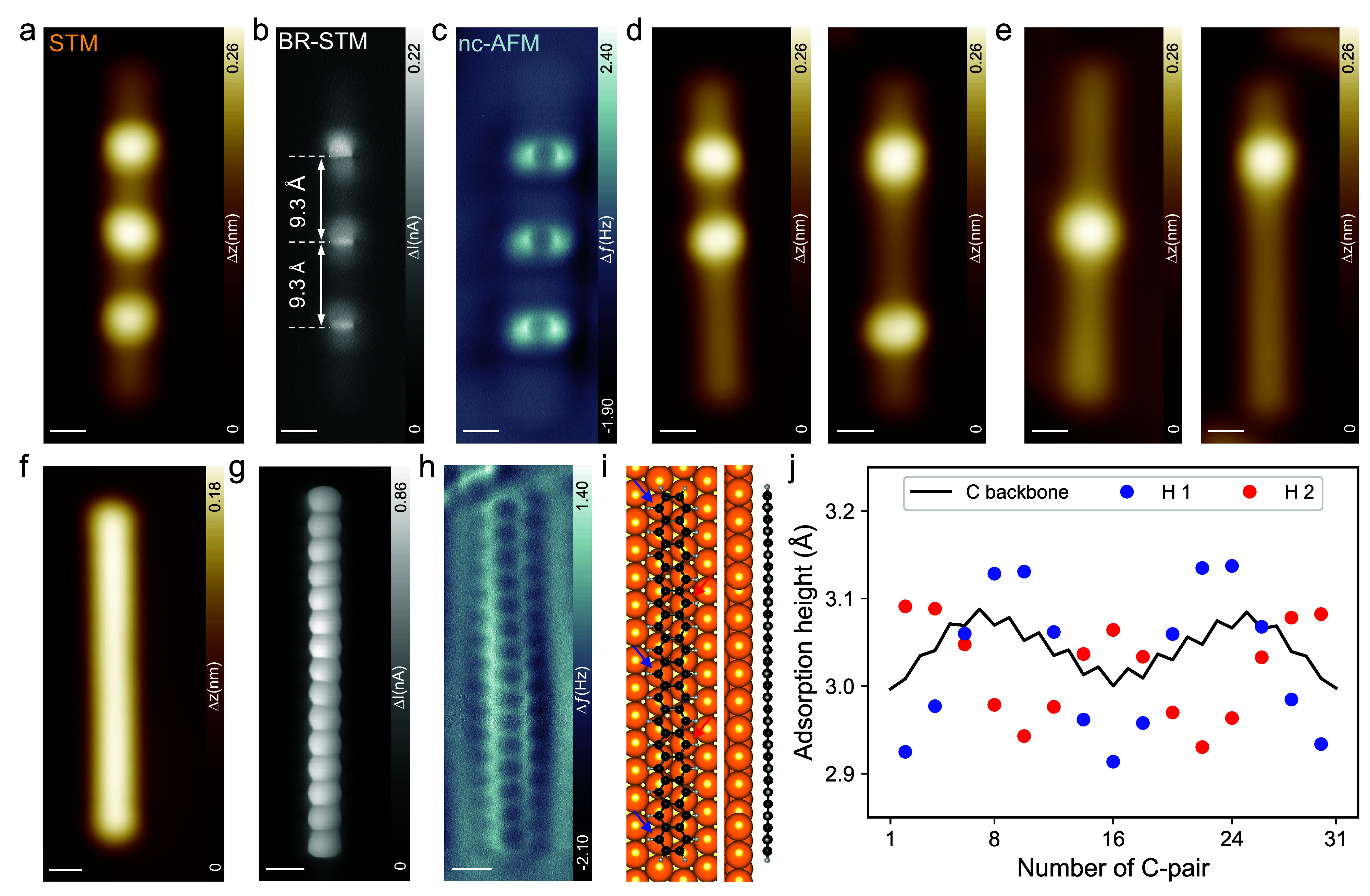
**On-surface generation of 15ac by STM tip manipulation**. (a) STM, (b) BR-STM and (c) nc-AFM image of the precursor molecule, showing the three equally spaced etheno bridges. (d) STM images of the intermediates after removing one or (e) two etheno bridges by STM tip manipulation. (f) STM, (g) BR-STM and (h) nc-AFM images of **15ac**. (i**)** DFT optimized model of **15ac** on Au(111). (j**)** the adsorption height of carbon atoms at the edges derived from the model in (i). Scale bar: (a-h) 0.4 nm for all images. Scanning parameters: (a, d, e, f) V_s_ = 0.15 V, I_t_ = 10 pA; (b, c, g, h) V_s_ = 2 mV.

The trietheno-bridged pentadecacene precursor **1** (three isomers, see Scheme 1) was synthesized in solution (see Methods and Supporting Information) by a sequence of Diels–Alder reactions involving arynes and dienes followed by dehydrogenation with 3,4-dichloro-5,6-dicyano-1,4-benzoquinone (DDQ) (Scheme 1a). For the experiment conducted in UHV, the stable precursor **1** was vapor deposited onto the clean Au(111) surface kept at 140 K. Subsequent STM imaging at 4 K reveals linear molecules with three protrusions (Figure 1a and Supplementary Figure S1), which were assigned to the /111/ isomer of precursor **1** in its stretched-out adsorption conformation. In contrast to the trietheno-bridged **13ac** precursor,^24^ which preferentially adopts an edge-on adsorption configuration, the **15ac** precursors are only rarely found in this geometry (Supplementary Figure S2), while the stretched-out /111/ configuration appears more frequently. This can be understood by comparing the structures of the trietheno-bridged /111/ precursors. The **15ac** precursor has an additional benzene ring in the inner connections between the etheno-bridges compared to the **13ac** precursor, resulting in a larger opening between the ends. Consequently, it can bend open more easily and transition more readily into the stretched-out conformation. This is further evidenced by the ∼23 kJ/mol lower energy required for the deformation of the **15ac** precursor compared to the **13ac** precursor (Supplementary Table S1). The stretched-out **15ac** precursors are surrounded by smaller molecules, which we attribute to the partial fragmentation of the precursor molecules at the required high sublimation temperature of ∼710 K. Figure 1a shows the STM image of the intact **15ac** precursor molecule **1b** isolated by lateral STM tip manipulation. The corresponding high-resolution bond-resolved STM image (Figure 1b) and the constant current nc-AFM image (Figure 1c) obtained with a CO-functionalized tip^27^ confirm the presence of three equally spaced etheno bridges with a distance of ∼9.3 Å, which is in good agreement with the DFT-optimized adsorbate (∼9.6 Å). This distance is shorter than expected from the gas phase C-shaped/111/precursor (∼10.1 Å in DFT) due to the remaining out-of-plane distortion. The length of the distorted/111/isomer on Au(111) is ∼36.3 Å as measured by constant current STM (Supplementary Figure S3), which is again in good agreement with the value for the DFT-optimized adsorbate of ∼36.4 Å. Having successfully deposited **1**, we next performed sequential tip manipulation on the stretched-out isomer to remove the three etheno bridges, as we have developed previously for **13ac**.^24^Figure 1d shows a series of STM images of all possible intermediates after the successful removal of one or two etheno-bridges from **1** by applying a pulse voltage of 2.5–3.0 V, which yields the dietheno-bridged and monoetheno-bridged intermediates. Figure 1f shows the STM image of the final product, presumably **15ac**, after removing all three etheno-bridges. To ultimately confirm the successful generation of **15ac**, the CO-functionalized tip is utilized to achieve submolecular resolution. In the BR-STM image (Figure 1f) and the nc-AFM image (Figure 1g), all the 15 six-membered rings are unambiguously resolved, confirming the successful generation of **15ac**. The DFT-optimized structure demonstrates that **15ac** on Au(111) (Figure 1i) is bent toward the surface in the center as well as at both ends, with the two long edges showing different adsorption heights relative to each other, as can be clearly seen from the height profiles in Figure 1j. Such nonplanar adsorption configuration has also been observed for **13ac**.^24^ Again, we attribute this bending to an incommensurability between the carbon backbone of **15ac** and the Au(111) substrate, leading to an anisotropy in dispersion attraction and thus to different adsorption heights. The decomposition of the adsorption energy confirms that **15ac** is bound to Au(111) purely by dispersion forces, even with repulsive contributions from electronic orbital interactions (Supplementary Table S2). This is in line with the experimentally observed extremely high mobility of the generated **15ac**, which suggests weak interaction with the Au(111) substrate.

**Figure 2:**
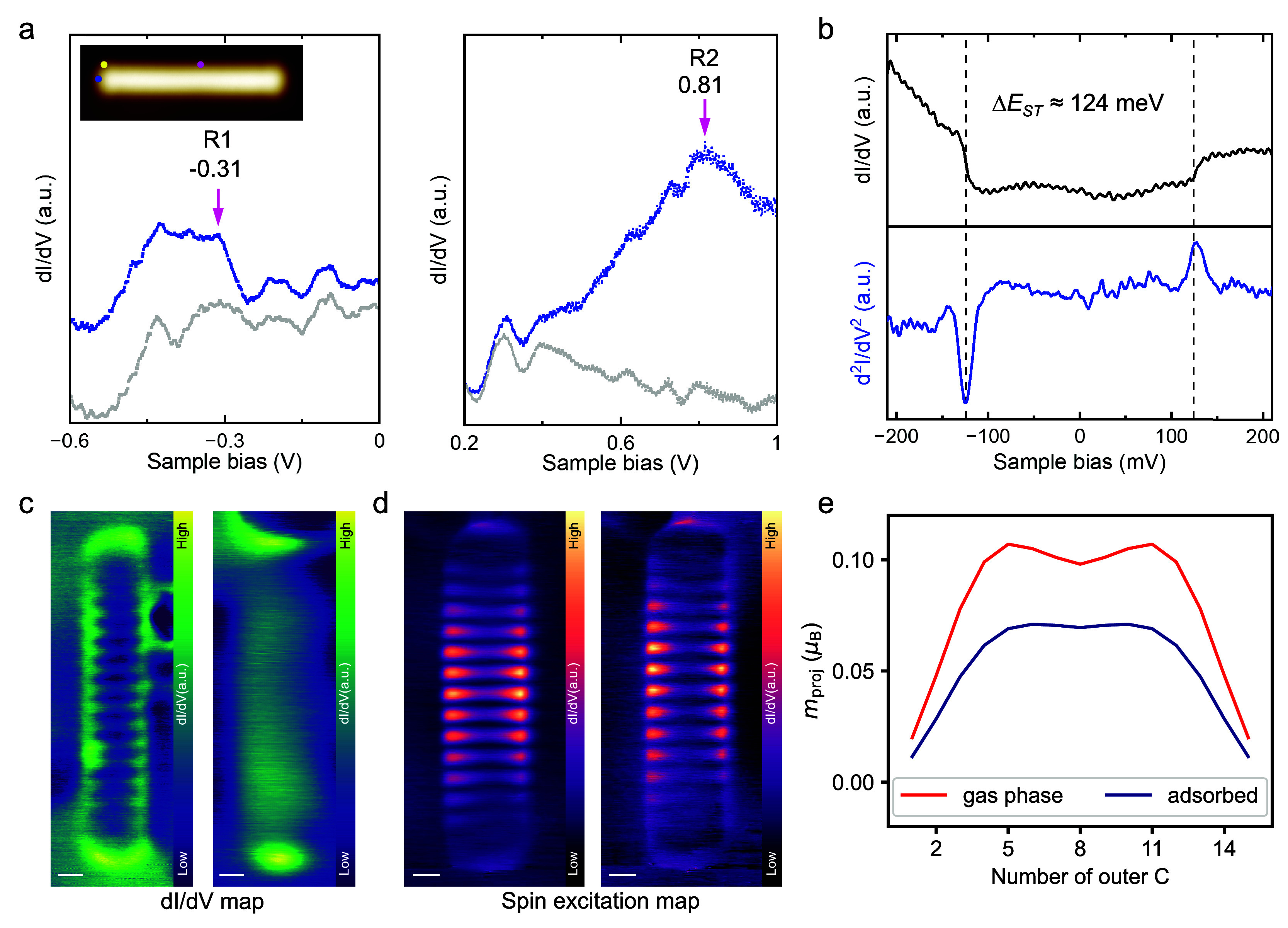
**Electronic properties of 15ac**. (a) dI/dV curves measured at positions marked in the inset (yellow dot for occupied state and blue dot for unoccupied state); two resonance peaks are identified as HOMO and LUMO (purple arrows). The gray curves are acquired from the bare Au(111) surface. (b) Low-bias dI/dV curve (top) taken at position marked in (a) (magenta dot) and its corresponding numerically calculated inelastic tunneling spectrum (d^2^I/dV^2^), showing the symmetric inelastic peaks centered around zero bias. (c) dI/dV maps taken at resonance peaks R1 (left) and R2 (right) in (a). (d) Spin excitation maps recorded at −124 mV (left) and +124 meV (right). (e) Atom-projected magnetization of the outer major-spin carbon atoms for **15ac** in the gas phase (blue) and **15ac** adsorbed on Au(111) (red). Scale bar: (c, d) 0.3 nm. Scanning parameters: (c) (left) V_s_ = −0.3 V and (right) V_s_ = 0.8 V, I_t_ = 100 pA. (d) (left) V_s_ = −124 mV and (right) V_s_ = 124 mV, I_t_ = 250 pA; (a) V_rms_= 15 mV, (b) V_rms_ = 8 mV.

We then performed STS measurements to probe the electronic properties of **15ac**. Figure 2a shows the wide range dI/dV curves measured on the occupied and unoccupied regions, where two peaks at −0.31 and 0.81 V are identified. We assign the two peaks to the LUMO and HOMO related resonance states, indicating an STS transport gap of around 1.12 eV. This value is slightly larger than the value previously determined for **11ac** and **13ac** (both 1.09 eV).^17,24^ Apparently, the monotonous decrease that was observed for the smaller acenes up to 11ac is no longer present. However, there is also no increase in the gap as reported for **12ac** (to 1.4 eV).^18^ The STS transport gap therefore no longer seems to change significantly with length, which is rather unexpected. For a theoretical treatment, we use spin-polarized DFT calculations to describe the electronic structure of **15ac** in the gas phase and in the adsorbed state on Au(111). In the gas phase, the antiferromagnetic (afm, also open-shell singlet or (poly-)radical) state is more stable than the nonmagnetic (nm) state by 8 kJ/mol (Supplementary Table S2).^28^ The adsorption was modeled using a slab ansatz to get a qualitative idea of the influence of the Au(111) substrate on the electronic state of the molecule. The electronic screening between the surface and the molecule should reduce the effects of static correlation, justifying the use of a single-reference method.^29−31^ Similar to **13ac**, the calculations show that the substrate leads to a reduction of the HOMO–LUMO gap, for **15ac** by 19% (Supplementary Figure S4). Interestingly, the relative reduction is smaller than for **13ac**(24) (25%, see also Supplementary Table S3). This could be an indication of the experimental observation made here: although the “real” gas phase gap presumably decreases from **13ac** to **15ac** (Supplementary Note 8), the difference in the influence of the Au(111) substrate counteracts this trend, resulting in the gap of the adsorbed molecules being almost the same. The experimental dI/dV mapping at the resonance peaks R1 and R2 shown in Figure 2c reveal the typical features also observed for smaller acenes.^17,18,22,24^

Notably, a spin excitation feature around the Fermi energy, which is the characteristic of a singlet open-shell electronic structure,^32^ has also been observed (Figure 2b). From the numerically obtained inelastic tunneling spectra (d^2^I/dV^2^), the singlet–triplet gap is determined to be 124 mV. Such spin excitation feature has recently been observed for **13ac** generated from a hydroacene precursor,^22^ while it was absent for **13ac** produced by STM tip manipulation,^24^ and also absent for undecacene (**11ac**)^17^ and dodecacene (**12ac**),^18^ for which open-shell ground states had been theoretically predicted.^4,5,26,28^ Notably, the small singlet–triplet excitation energy Δ*E*_ST_ observed here agrees with previous experimental observation for **13ac** (126 mV).^22^ In the case of **13ac**, it had been proposed that the open-shell character is significantly reduced by the adsorption on the Au(111) surface.^24^ Additionally, such a small Δ*E*_ST_ value has been well explained by many-body ab initio calculations, which show that the dynamic electron correlation and the influence of the substrate cause a significant reduction in Δ*E*_ST_.^22^ Our theoretical calculations also show a reduction in the magnetization of **15ac** upon adsorption by 36% (Figure 2e). This value is larger than the value previously calculated for **13ac**(24) (26%, see also Supplementary Table S4) and shows an opposite trend to the HOMO–LUMO gap: While the reduction in the gap is smaller for **15ac** (19% vs 25%), the reduction in magnetization is larger for **15ac** (36% vs 26%) Another noteworthy feature is revealed when looking at the distribution of the magnetization of **15ac** in the gas phase (Figure 2e). It shows two maxima, indicating a tetraradical character of the open-shell singlet. This polyradical (beyond diradical) character has also been predicted for acenes of this length in other theoretical works,^4,5,26,28,33,34^ although the degree of polyradical character strongly depends on the level of theory (see Trinquier et al.^4^ for discussion of **15ac** and Tönshoff and Bettinger^7^ for a comparison).

However, adsorption on the Au(111) surface not only reduces the amount of magnetization, as already mentioned, but also changes the distribution. Thus, the tetraradical character of **15ac** is reduced upon adsorption to a larger diradical contribution. Interestingly, the change is dependent on the adsorption site, as shown by the difference between the two long zigzag edges (Supplementary Figure S5). The decrease and change in polyradical character of the long acene upon adsorption is another interesting observation that once again emphasizes the importance of including the substrate in the interpretation of the measured electronic structure. It is worth noting that spin-polarized DFT reaches its methodological limits with such long acenes due to a lack of static correlation (Supplementary Note 8), and conclusions should only be viewed as qualitative. However, since the explicit inclusion of the substrate in wave function-based multireference methods is not computationally feasible, it is still the most convenient method available to investigate the effect of the substrate. In line with the theoretical predictions (see Supplementary Figure S5c), the experimental spin excitation maps (Figure 2d) show noticeable ladder features, which are most pronounced at the center and become weaker near the ends of the molecule similar to **13ac**,^22^ contrasting with the dI/dV maps of HOMO and LUMO (Figure 2c) where the states are located at both the edges and the ends.

**Figure 3:**
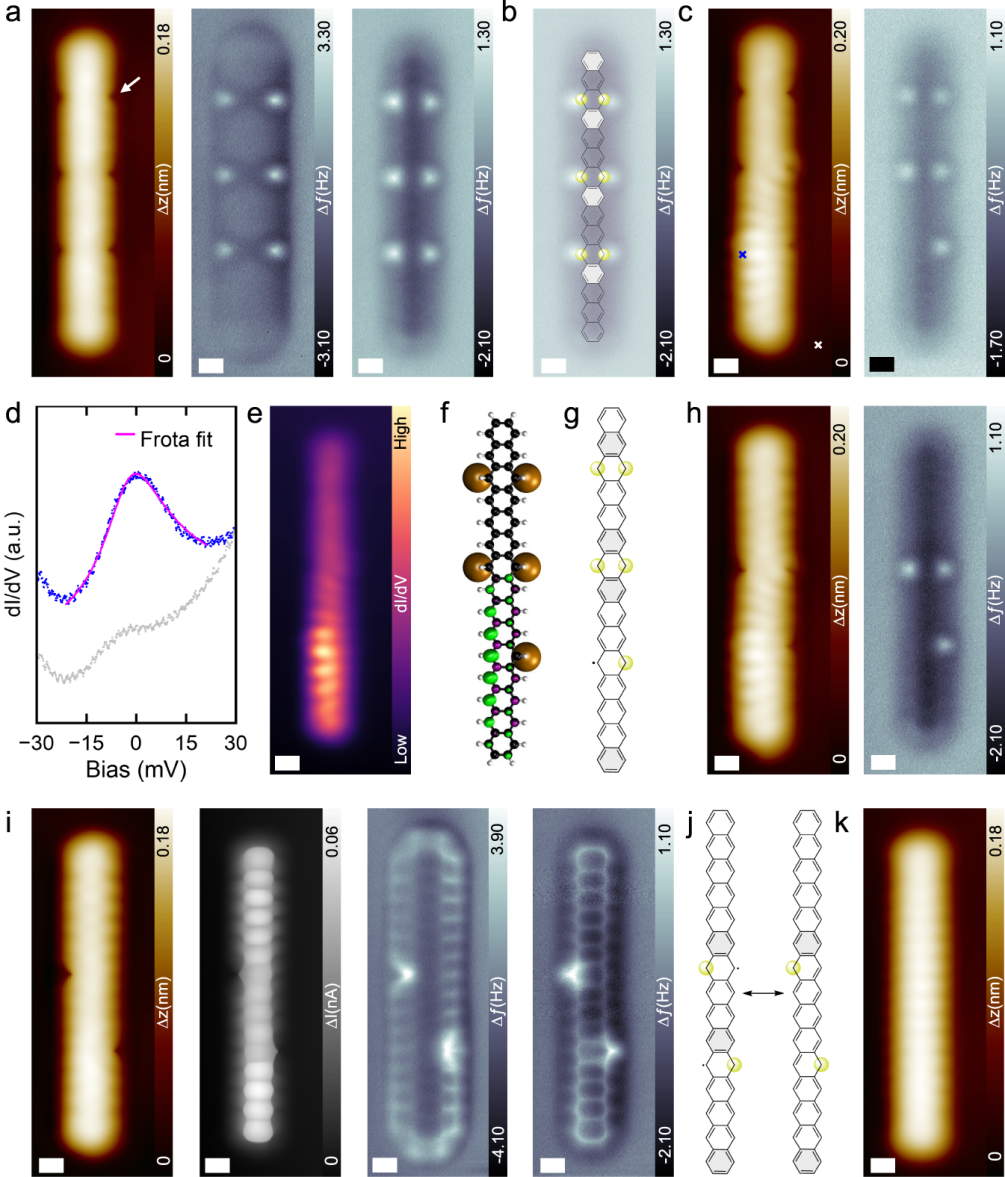
**Manipulation and Characterization of a Au-15ac complex**. **(**a) (Left to right) STM, CC-nc-AFM and CH-nc-AFM of the **6Au-15ac** complex. (b) The same CH-nc-AFM image as shown in (a), overlaid with the corresponding chemical model. (c) STM and CH-nc-AFM image of the **5Au-15ac** complex. (d) (left) Low-bias dI/dV spectra taken at positions marked by the crosses in (c). (e) dI/dV map taken at 2 mV. (f) Spin density (ρ_α_-ρ_β_) distribution of the **5Au-15ac** complex. (g) The corresponding chemical structure of **5Au-15ac**. (h) STM and CH-nc-AFM image of the **3Au-15ac** complex. (i) (Left to right), STM, BRSTM, CC-nc-AFM and CH-nc-AFM image of the **2Au-15ac** complex. (j) Possible resonance structures of the **2Au-15ac** complex. (k) STM image of a **15ac** after removing all the Au adatoms. Scale bars: 0.3 nm for all experimental images. Scanning parameters: STM images: V_s_ = 10 mV, I_t_ = 5 pA; BR-STM and CH-nc-AFM images: V_s_ = 5 mV; CC-nc-AFM images: V_s_ = 20 mV, I_t_ = 8 pA. V_rms_ = 8 mV.

Interestingly, increasing the sublimation temperature to 750 K can induce the thermal cleavage of the etheno bridges during the deposition process, resulting in the direct generation of **15ac**. The directly generated **15ac** lands on the Au(111) surface at 150 K, which enables its reaction with readily available gold atoms. Figure 3a shows the STM image of a linear product with six noticeable indentations at the edges at low bias, and pronounced protrusions at high bias (Supplementary Figure S6). The corresponding nc-AFM images taken at constant current and constant height mode show consistent bright protrusion at these sites (see also Supplementary Figure S7). Such features have previously been observed for adsorbed open-shell carbon nanostructures and have been attributed to metal adatoms,^19,21,35^ which stabilize the reactive species. We confirmed the presence of a metal complex by detailed STM tip manipulations and visualizing the reversible nature of the complexation process (see Supplementary Figure S8–S11 and the related text for more details). In the next step, we used the STM tip to successively eliminate the adatoms. Specifically, the STM tip was positioned above the indentation feature, and the tip was retracted by several hundred picometers after switching off the feedback loop, then the bias was ramped until an abrupt change of the tunneling current was observed. Subsequent imaging revealed a significant change in the appearance of the molecule, as shown in Figure 3c. Remarkably, the adatom on the opposite side of the molecule remained unaffected, as can be clearly seen in the corresponding nc-AFM image (Figure 3c). This observation is in contrast to previous observations on acene metal complexes, for which the two metal atoms are removed simultaneously at both sides of the molecule.^19^ The abstraction of a single gold adatom leaves an unpaired π electron, which is evidenced by the occurrence of the Kondo effect. Specifically, we observe a pronounced zero-bias peak in low-energy dI/dV spectra acquired on the molecule shown in Figure 3d. The peak is well described by a Frota function, indicative of a Kondo resonance.^36,37^ Moreover, the constant height dI/dV map (Figure 3e) at low bias approximating the Kondo map demonstrates that the spins are localized in the lower part of the molecule and shows good agreement with the calculated spin density map in the gas phase (Figure 3f). These observations unambiguously evidence the presence of an unpaired electron (*S* = 1/2).^32,38^ The removal of the remaining gold adatoms can be achieved via a similar tip manipulation process. Figure 3h shows the STM image taken after attempting removal of the Au atom on the top left; however, the other Au atom that binds to the same hexagon was removed simultaneously, as can be observed from the nc-AFM image (Figure 3h, right), resulting in a **3Au-15ac** complex. By further repeating the manipulation protocol, we successfully removed one of the Au atoms at the center part of the complex (Figure 3i, left). One can easily notice the vanishing of the Kondo scattering feature at the bottom left in the STM images, and the intramolecular structures of the resulting **2Au-15ac** complex can be well captured by high-resolution imaging (Figure 3i, second image from left). Additionally, in the constant current nc-AFM image (Figure 3i, third image from left), the hydrogen atoms at the zigzag edge, including the sites where the Au atoms were released, are clearly identified, suggesting the successful removal of the Au atoms without damaging the **15ac**. Further STS measurements indicate the absence of low-bias features (Supplementary Figure S12), which thus suggests that the molecule is in a singlet state. Gas phase model calculations of **2Au-15ac** showed that closed-shell and open-shell singlet essentially have the same energy (Supplementary Figure S11). Therefore, the electronic ground state cannot be determined with certainty. It can only be ruled out that the molecule is in a triplet state, which is 17 kJ/mol higher in energy than the singlet state (Supplementary Figure S11). Further tip manipulation induces the simultaneous removal of the remaining two Au atoms, resulting in the formation of **15ac** (Figure 3k and Supplementary Figure S13). In some cases, a single atom that complexed with **15ac**, which is expected to hold the *S* = 1/2 ground state, is achieved, and its doublet character has been confirmed by further dI/dV measurement (Supplementary Figure S14). The observation of the spontaneous complexation of **15ac** with up to 6 Au atoms on the Au(111) surface (see Supplementary Figure S14 and S15 for more examples) reveals the high reactivity of the long acene. Furthermore, this indicates a significant contribution of the polyradical character to the ground state of **15ac**, as significantly more Au adatoms were bound than previously observed for **7ac**, **9ac** and **13ac**, where complexes with only 2 Au atoms were identified.^19,24^

## Conclusion

In conclusion, we have demonstrated the on-surface synthesis of pentadecacene (**15ac**) on the Au(111) surface achieved by a multistep removal of etheno groups from a trietheno-bridged precursor molecule, or the elimination of adatoms in a gold-pentadecacene complex spontaneously formed after thermally induced cleavages of the etheno-bridges. STS measurements of **15ac** revealed a transport gap of 1.12 eV, which is similar to the values of **11ac** and **13ac**. Spin-polarized DFT calculations showed that, upon adsorption, the HOMO–LUMO gap of **15ac** is reduced similarly to **13ac**, although the relative reduction is smaller than that for **13ac**. Furthermore, low-bias STS measurements revealed an open-shell singlet electronic state with an experimental singlet–triplet gap of 124 meV. Spin-polarized DFT calculations showed that the radical character of **15ac** is reduced upon adsorption. Interestingly, the polyradical contribution is also reduced, resulting in an increased diradical character on the surface. Complexation of up to 6 Au adatoms observed in the experiment supports the assumption of a polyradical character of **15ac**.

## Experimental and Computational Details/Methods

### Synthesis of the Precursor

#### General

Unless otherwise stated, all chemicals were either used as received from their respective commercial suppliers or purified according to literature recommendation.^39^ DCM and toluene were dried using a Braun SPS-800 solvent drying system. Dry MeCN and CHCl_3_ were purchased from Acros. Solvents for flash column and thin layer chromatography, including dichloromethane and *n*-hexane were all of HPLC grade quality. CsF and KF were vacuum dehydrated with a heat gun before use. Compounds **2**(40) and **5**(41) were prepared according to the literature. Thin layer chromatography was performed on fluorescence indicator marked precoated silica gel plates (0.040–0.063 mm). The reactions were carried out in an inert gas atmosphere using argon as protecting gas.

#### NMR Spectroscopy

^1^H and ^13^C NMR spectra were recorded on a Bruker Avance III HD 300 NanoBay, on a Bruker Avance III HD 400, on a Bruker Avance III HDX 600 or on a BRUKER Avance III HDX 700 instrument. Chemical shifts for ^1^H NMR are reported as δ relative to tetramethylsilane, the signals of residual CHCl_3_ at 7.26 ppm and of 1,2-dichlorobenzene at 6.93 and 7.19 ppm were used as internal references. Chemical shifts for ^13^C NMR are reported as δ relative to tetramethylsilane, the signals of CDCl_3_ at 77.16 ppm and of CD_2_Cl_2_ at 54.95 ppm were used as internal references. The following abbreviations were used to describe splitting patterns: s = singlet, m = multiplet.

#### Mass Spectrometry

High-resolution atmospheric pressure chemical ionization (APCI) spectra were obtained on a Bruker maxis 4G with a TOF mass analyzer.

#### Synthesis of 3-(Trimethylsilyl)-5,6,7,14,15,16-hexyhydro-6,15-ethenohexacen-2-yltrifluoromethanesulfonate (**4**)

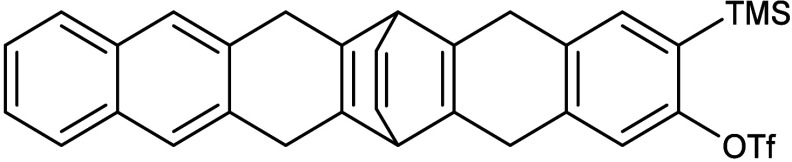



Diene **2** (85 mg, 300 μmol), bistriflate **3** (120 mg, 232 μmol) and CsF (150 mg, 987 μmol) were suspended in dry MeCN (12 mL) and heated at 45 °C for 19 h. The solvent was removed and 20 mL of H_2_O and 30 mL DCM were added. The organic phase was separated, and the aqueous phase was extracted once with 30 mL DCM. The solvent was removed in vacuo and the crude product was purified by column chromatography (silica gel, DCM:*n*-hexane 1:5). After removal of the solvent, 50 mg (87 μmol, 37%) of **4** was obtained as a yellow solid.

^**1**^**H NMR** (700 MHz, CDCl_3_) δ [ppm]: 7.73 (2H, m), 7.62 (2H, s), 7.38 (2H, m), 7.25 (1H, s), 7.09 (1H, s), 6.90 (2H, m), 4.35 (2H, m), 3.79 (4H, s), 3.63 (4H, m), 0.35 (9H, s).

^**13**^**C NMR** (176 MHz, CDCl_3_) δ [ppm]: 153.2, 140.6, 140.5, 139.7, 139.5, 138.4, 136.6, 134.0, 133.2, 132.4, 129.7, 127.2, 127.2, 126.9, 126.9, 125.2, 119.4, 54.4, 54.2, 33.5, 33.3, 32.7.

**HRMS (APCI)***m*/*z*: [M + H]^+^ calculated for C_32_H_30_F_3_O_3_SSi^+^ 579.16315; measured 579.16196.

***R***_**f**_**value** (silica gel, DCM:*n*-hexane 1:1): 0.80.

#### Synthesis of 6,7,8,10,11,12,14,15,16,23,24,25,27,28,29,31,32,33-Octadecahydro-7,32:11,28:15,24-triethenobenzo[*b*]tetraceno[2,3-*w*]decacene (**6**)

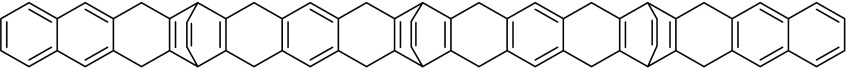



A suspension of triflate **4** (100 mg, 173 μmol), pentaene **5** (10.8 mg, 69 μmol), KF (121 mg, 2.08 mmol), 18-crown-6 (365 mg, 1.38 mmol), 30 mL dry DCM and 20 mL dry MeCN was stirred for 16 h at RT in the dark. The solvent was removed in vacuo and the crude product was filtered with a precolumn consisting of silica gel and DCM as solvent. The solvent was removed in vacuo and the crude product was purified by column chromatography (silica gel, DCM:*n*-hexane 1:2). After removal of the solvent, 16 mg (18 μmol, 26%) of **6** was obtained as a colorless solid.

^**1**^**H NMR** (400 MHz, CDCl_3_) δ [ppm]: 7.70 (4H, m), 7.59 (4H, s), 7.36 (4H, m), 6.82 (10H, m), 4.32 (4H, m), 4.23 (2H, m), 3.78 (8H, m), 3.50 (16H, m).

^**13**^**C NMR** (176 MHz, CDCl_3_) δ [ppm]: 140.5, 140.4, 139.5, 139.4, 133.5, 132.3, 132.0, 131.9, 128.7, 127.2, 126.9, 125.2, 54.5, 54.5, 33.3, 32.9.

A mass spectrum of the compound could not be obtained due to its instability.

***R***_**f**_**value** (silica gel, DCM:*n*-hexane 1:1): 0.68.

#### Synthesis of 7,11,15,24,28,32-Hexahydro-7,32:11,28:15,24-triethenobenzo[*b*]tetraceno[2,3-*w*]decacene (**1**)

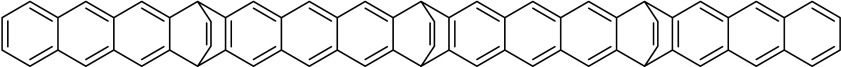



Compound **6** (25 mg, 29 μmol) and 2,3-dichloro-5,6-dicyanoquinone (DDQ) (131 mg, 577 μmol) were added to 18 mL of dry CHCl_3_ and the mixture was stirred for 3.5 h at RT in the dark. To the reaction mixture, 4 mL of a saturated NaHCO_3_ solution was added and the mixture was stirred for 10 min at RT. The organic phase was separated, and the aqueous phase was extracted twice, each time with 20 mL CHCl_3_. The solvent was removed in vacuo and the crude product was purified by column chromatography (silica gel, DCM:*n*-hexane 2:3). After removal of the solvent, **1** was obtained as a colorless solid with a yield of 8% (2 mg, 2.33 μmol).

^**1**^**H NMR** (700 MHz, CS_2_: CDCl_3_, 10:1) δ [ppm]: 8.23–8.07 (8H), 7.89 (4H, m), 7.80–7.74 (12H), 7.36 (4H, m), 7.01 (6H, m), 5.32–5.24 (6H, m).

^**13**^**C NMR** (150 MHz, CDCl_3_) δ [ppm]: 141.3, 141.2, 140.9, 140.8, 140.7, 137.9, 137.8, 137.7, 132.0, 132.0, 131.8, 130.9, 130.8, 130.8, 128.2, 128.2, 125.8, 125.8, 125.3, 125.1, 125.1, 121.1, 53.5, 50.2.

**HRMS (APCI)***m*/*z*: [M + H]^+^ calculated for C_68_H_41_^+^ 857.32028; measured 857.31932. We observed in addition a signal at *m*/*z* 631.24102. This could be due to an undecacene derivative resulting from the reaction of **2** and **3** by 2-fold aryne generation and Diels–Alder reaction though we have not obtained evidence for its formation in prior steps of the synthesis.

***R***_**f**_**value** (silica gel, DCM:*n*-hexane 2:3): 0.29.

### On-Surface Synthesis

#### Sample Preparation

The atomically clean Au(111) (MaTecK) surface was obtained by cycles of argon-ion sputtering and annealing at 800 K. The trietheno-bridged precursor was vapor-deposited from a standard Knudsen cell heated to 710 or 750 K, while the Au(111) substrates were held at 140 K. Carbon monoxide (CO) molecules were dosed onto the sample surfaces for tip modification.

#### Scanning Probe Microscopy Characterization

Experiments were performed by using an ultrahigh vacuum low temperature-scanning tunneling microscope (UHV LT-STM, Scienta Omicron) with a base pressure better than 1 × 10^–10^ mbar. All the STM images were acquired with constant current or constant height mode by using an electrochemically etched tungsten tip at 4.2 K. All given voltages were applied to the sample with respect to the tip. Nanotec Electronica WSxM software was used to process the images.^42^ Constant current mode nc-AFM measurements were performed at 4.2 K with tungsten tips placed on a qPlus tuning fork sensor^43^ driven at its resonance frequency (26500 Hz) with a constant amplitude of ∼70 pm. The tips were functionalized with a single CO molecule at the tip apex picked up from the Au surface after dosing CO. The Δz was positive (negative) when the tip–surface distance was increased (decreased) with respect to the STM set point at which the feedback loop was opened. The dI/dV spectra were recorded using a lock-in amplifier with a modulation frequency of 579 Hz and an amplitude of 8–15 mV. dI/dV maps were collected in constant-current mode.

### Computational Investigations

All calculations were carried out using density functional theory with periodic boundary conditions implemented in the Vienna Ab Initio Simulation Package (VASP) Version 5.4.4.^44−47^ The projector-augmented wave formalism^48,49^ and the PBE functional^50,51^ together with the third-generation van der Waals dispersion correction by Grimme (DFT-D3) with Becke-Johnson damping^52,53^ were used. The Au(111) surface was modeled using a 4-layered slab with the previously determined Au bulk lattice parameter of 4.101 Å.^24^ 13ac, 15ac, and the stretched-out linear/111/isomer were placed in a 15 × 5 supercell at the same adsorption site as previously established for 13ac.^24^ Various configurations were tested for the Au adatoms-15ac complexes in the 15 × 5 and in the (9√3 × 3√3)-R30° supercell (for more details see Supplementary Section 14). In all cases, the bottom two layers were frozen in their bulk position, while the top two layers and the molecule were freely optimized. A plane-wave cutoff energy of 400 eV, a 1 × 3 × 1 k-grid, a vacuum layer of 20 Å and the second-order Methfessel-Paxton smearing method with a smearing width of 0.01 eV were employed as obtained by previously conducted convergence studies.^24^ The convergence criterion of the self-consistent field was set to 10^–5^ eV and the structural optimization convergence criterion was set to 10^–2^ eV/Å. The spin-polarized DFT calculations were initialized by assigning spin up (α) to the outer carbon atoms on one side of the nonmagnetic structure and spin down (β) on the other side of this structure. The projected density of states (PDOS) and projected magnetization were directly obtained from VASP. However, the k-grid was improved to 2 × 6 × 1 to avoid artifacts.

## Data Availability

Computational data underlying this manuscript are freely accessible at the NOMAD repository under 10.17172/NOMAD/2024.11.19-1. Spectral data underlying this study are available in the published article, in its Supporting Information, and openly in RADAR4Chem at 10.22000/xbhtmsgkufv96vpg.

## Supporting Information Available

Synthetic procedures and spectroscopic data for all new compounds, including images of NMR spectra, additional SPM data, additional computational results (PDF)
