# Supplementary material for: On-Surface Synthesis and Characterization of Pentadecacene and Its Gold Complexes
Source: J Am Chem Soc. 2025 Jan 17;147(6):4862–70. doi: 10.1021/jacs.4c13296 (PMC11827000; doi:10.1021/jacs.4c13296)
Supplement: Supplementary file 1 — ja4c13296_si_001.pdf [file ja4c13296_si_001.pdf]

Supporting Information

On-Surface Synthesis and Characterization of Pentadecacene and its Gold Complexes

Zilin Ruan[1,†], Jakob Schramm[2,†], John B. Bauer[3,†], Tim Naumann[1], Laurentia V. Müller[3], Felix Sättele[3], Holger F. Bettinger[3]*, Ralf Tonner-Zech[2]*, J. Michael Gottfried[1]*

[1]*Philipps-Universität Marburg, Fachbereich Chemie, Hans-Meerwein-Str. 4, 35032 Marburg, Germany, michael.gottfried@chemie.uni-marburg.de*

[2]*Universität Leipzig, Fakultät für Chemie und Mineralogie, Wilhelm-Ostwald-Institut für Physikalische und Theoretische Chemie, Linnéstraße 2, 04103 Leipzig, Germany, ralf.tonner@uni-leipzig.de*

[3]*Universität Tübingen, Institut für Organische Chemie, Auf der Morgenstelle 18, 72076 Tübingen, Germany, holger.bettinger@uni-tuebingen.de*

† These authors contributed equally and share the first authorship.

1. Overview STM image after depositing the precursor 1 onto the Au(111) surface

In contrast to the shorter trietheno precursor for tridecacene (13ac)[1], which shows a dominating edge-on adsorption geometry, here the intact precursor shows mainly a linear stretched-out configuration.

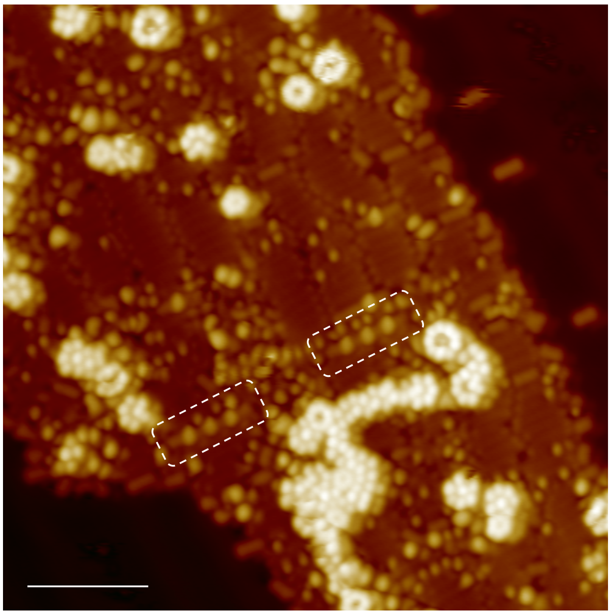

Figure S1. Large-scale STM image of Au(111) surface after deposition of the precursor 1. Two intact precursors are indicated by dashed rectangles. Scale bar: 5 nm. Scanning parameters: $V_s = 0.15$ V, $I_t = 20$ pA.

2. Edge-on adsorbed precursor molecules on the Au(111) surface

The edge-on adsorbed molecules are rarely found on the surface; however, they are stabilized by molecular fragments generated due to the high sublimation temperature needed.

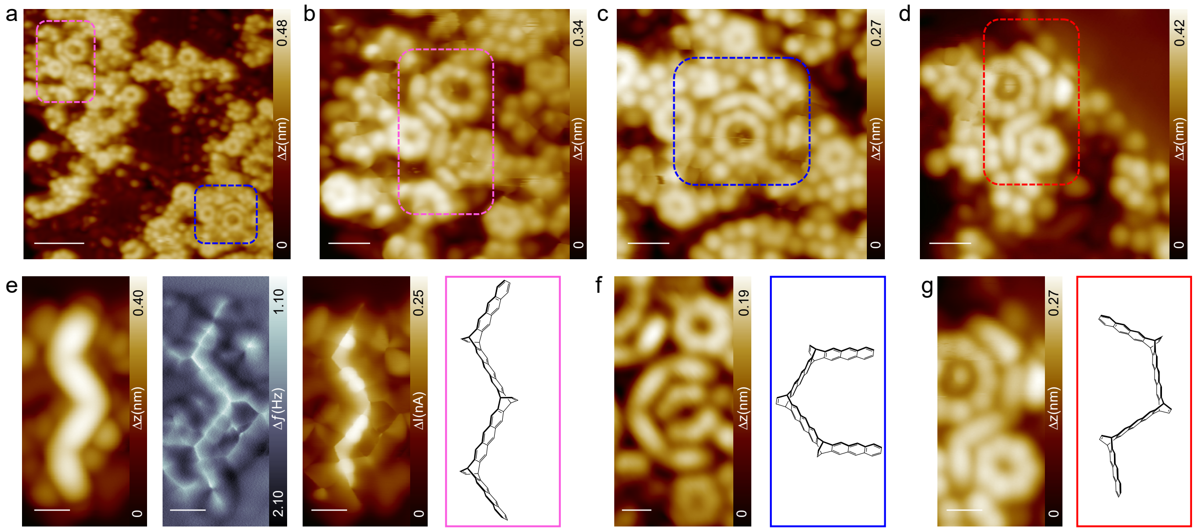

Figure S2. Edge-on adsorbed isomer on Au(111). (a) Large-scale STM image showing two intact edge-on molecules marked by colored rectangles. (b-d) STM images of the *M*-shape, *C*-shape and *S*-shape edge-on isomers. (e) (left to right) STM, CC-nc-AFM, simultaneously obtained BR-STM image and the corresponding chemical structure of the *M*-shape isomer, respectively. (f) Zoom-in STM image and the corresponding chemical structure of *C*-shape isomer. (g) Zoom-in STM image of the *S*-shape isomer and its chemical structure. Scale bar: (a) 3 nm; (b-d) 1 nm; (e, g) 0.6 nm; (f) 0.5 nm. Scanning parameters: (a-d, f-g) $V_s = 0.15$ V, $I_t = 15$ pA; (e) $V_s = 0.15$ V, $I_t = 50$ pA.

3. Decomposed adsorption energies of the stretched-out linear /111/ precursors

We decompose the adsorption energy to get more detailed information about the nature of the adsorbate-surface bond (see also Scheme S1). In general, the adsorption energy is defined as the difference between the energy of the optimized adsorbate-surface complex and the energy of the optimized molecule and the optimized surface.

$$E_{\mathrm{ads}} = E_{\mathrm{asc}}^{\mathrm{opt}} - \left(E_{\mathrm{mol}}^{\mathrm{opt}} + E_{\mathrm{surf}}^{\mathrm{opt}}\right)$$

One can now define the preparation energy as the difference between the respective fragment frozen in the adsorbate-surface complex (asc) geometry and the optimized fragment. It is a quantity that provides information on how strong the deformation is due to the formation of the bond.

$$E_{\mathrm{prep}}(\mathrm{surf}) = E_{\mathrm{surf}}^{\mathrm{frz}} - E_{\mathrm{surf}}^{\mathrm{opt}}$$

$$E_{\mathrm{prep}}(\mathrm{mol}) = E_{\mathrm{mol}}^{\mathrm{frz}} - E_{\mathrm{mol}}^{\mathrm{opt}}$$

Furthermore, one can define the interaction energy between the fragments frozen in the optimized geometry. It is defined as the difference between the energy of the adsorbate-surface complex and the energy of the frozen molecule and frozen surface. Since we used the additive DFT-D3(BJ) dispersion correction, we can apply this independently to the D3-dispersion energy (DE) and the Kohn-Sham energy (KS).

$$E_{\mathrm{int}}(\mathrm{disp}) = DE_{\mathrm{asc}}^{\mathrm{opt}} - \left(DE_{\mathrm{mol}}^{\mathrm{frz}} + DE_{\mathrm{surf}}^{\mathrm{frz}}\right)$$

$$E_{\mathrm{int}}(\mathrm{elec}) = KS_{\mathrm{asc}}^{\mathrm{opt}} - \left(KS_{\mathrm{mol}}^{\mathrm{frz}} + KS_{\mathrm{surf}}^{\mathrm{frz}}\right)$$

In the end, the adsorption energy can be recovered by adding up all these contributions. Additionally, the individual fragment preparation energies can be combined into a total preparation energy and the individual interaction energies into a total interaction energy.

$$E_{\mathrm{ads}} = E_{\mathrm{prep}}(\mathrm{mol}) + E_{\mathrm{prep}}(\mathrm{surf}) + E_{\mathrm{int}}(\mathrm{disp}) + E_{\mathrm{int}}(\mathrm{elec}) = E_{\mathrm{prep}} + E_{\mathrm{int}}$$

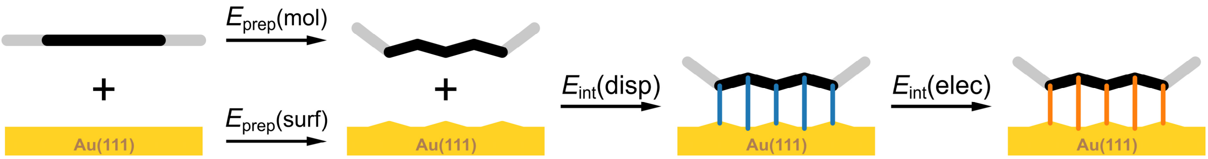

Scheme S1. **Schematic representation of the decomposition of adsorption energy used.**

Applying this decomposition to the /111/ precursor of **15ac** and comparing it to the /111/ precursor of **13ac** (Table S1), it is noticeable that the adsorption energy of **15ac** is more negative (ΔE_{ads} = -113 kJ mol^{-1}). The main reason for this is an increase in dispersion interaction (ΔE_{int}(disp) = -95 kJ mol^{-1}), simply because the **15ac** precursor contains two more benzene rings. This becomes evident when looking at the dispersion energy per atom (84 atoms for **13ac**, 96 atoms for **15ac**), which is in both cases essentially the same with 8 kJ mol^{-1}. However, it can also be seen that the preparation energy for the **15ac** precursor is lower (ΔE_{prep}(mol) = -23 kJ mol^{-1}), resulting from the fact that the precursor has a larger opening between the ends, which leads to an easier bending of the backbone.

Table S1. Decomposed Adsorption Energies for stretched-out /111/ precursors of 15ac and 13ac, as well as the difference between them (Δ). Energies are given in kJ mol^{-1}.

	/111/ for **15ac**	/111/ for **13ac**[a]	Δ(**15ac-13ac**)
E_{ads}	-616	-503	-113
E_{int}	-742	-650	-92
E_{int}(disp)	-766	-671	-95
E_{int}(elec)	24	21	3
E_{prep}	126	147	-21
E_{prep}(mol)	114	137	-23
E_{prep}(surf)	12	10	2

[a] Values from ref.[1]

4. Planarization of the stretched-out molecule after removing the etheno-bridges

The etheno-bridges induce out-of-plane distortion of the linear stretched-out molecule, and further planarization of the annulated rings are observed after tip manipulation.

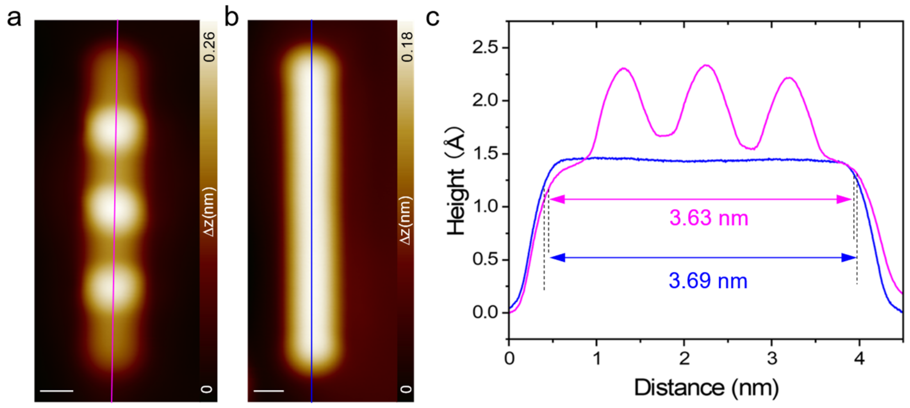

Figure S3. **Comparison of the length of the intact stretched-out precursor and 15ac.** (a) stretched-out precursor and (b) **15ac** and (c) their lengths measured along the dashed lines in (a) and (b), respectively. Scale bars: (a, b) 0.7 nm. Scanning parameters: (a, b) $V_s = 0.15$ V, $I_t = 10$ pA.

5. Decomposed adsorption energy of 15ac

Computationally, two solutions for the electronic states can be found for **15ac** in the gas phase and on the surface: an antiferromagnetic (afm) and a non-magnetic (nm) solution (Table S2). Note that the nm solution is a consequence of a closed-shell treatment and presumably not physically relevant. This is confirmed by the fact that in the gas phase, the afm state is more stable than the nm state by 8 kJ mol^{-1}. Upon adsorption, the relative stability of the state decreases, so it is only 3 kJ mol^{-1} more stable than the nm state. The reason for this is a less stable adsorption energy for the afm state by 5 kJ mol^{-1}. The decomposition of the adsorption energy reveals that the main contribution is an increase in electronic repulsion (E_{int}(elec)) by 4 kJ mol^{-1}, which is similar to the results of **13ac**[1].

Table S2. Relative Energies and decomposed Adsorption Energies for antiferromagnetic (afm) and non-magnetic (nm) 15ac, as well as the difference between them (Δ). Energies are given in kJ mol^{-1}.

	**15ac**-afm	**15ac**-nm	Δ(afm-nm)
E_{rel}(gas)	-8	0	
E_{ads}	-751	-756	+5
E_{int}	-764	-770	+6
E_{int}(disp)	-809	-811	+2
E_{int}(elec)	45	41	+4
E_{prep}	13	14	-1
E_{prep}(mol)	6	7	-1
E_{prep}(surf)	7	7	0
E_{rel}(ads)	-3	0	

6. Projected density of states of adsorbed 15ac

The projected density of states (PDOS) of the molecule reveals a relative reduction in the HOMO-LUMO gap upon adsorption by 19% for **15ac** (Figure S4a, see also Table S3). This is smaller than the previously determined value for **13ac** (25%).

A reduction in HOMO-LUMO gap is only found for the afm state of **15ac**. Similar to **13ac**, the nm state does not show a significant reduction[1]. Instead, it shows a slight increase by 9% (Figure S4b, see also Table S3).

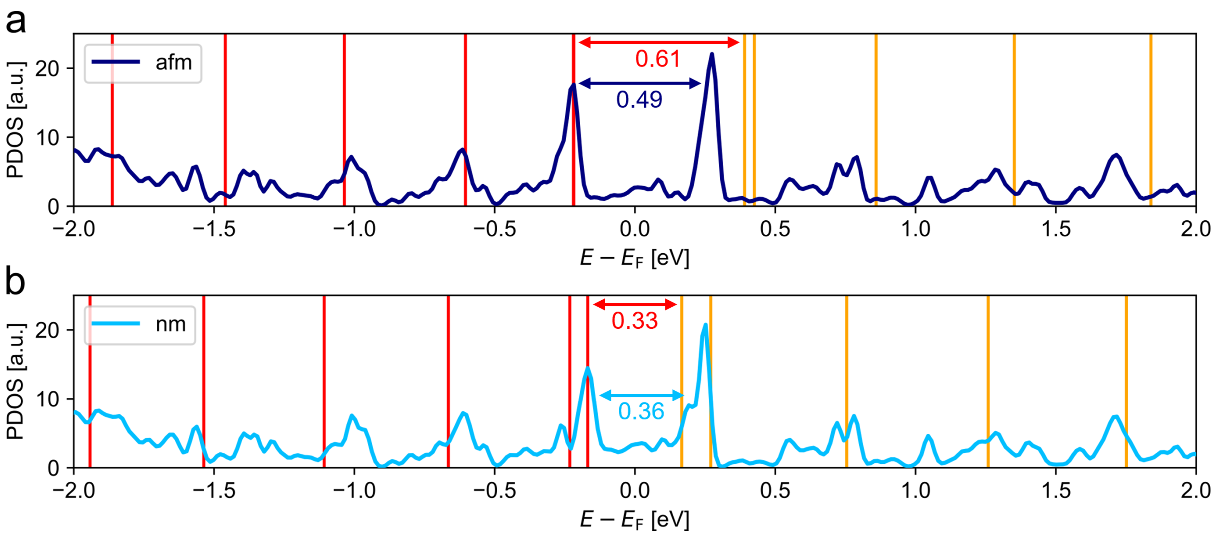

Figure S4. **Projected density of states of adsorbed 15ac.** (a) **15ac** in the antiferromagnetic (afm) state and (b) in the non-magnetic (nm) state with marking of the occupied (red) and virtual (orange) orbital energies of the respective state in gas phase. Only the HOMO energies of the gas phase molecules were aligned with the first peaks in the PDOS.

7. Comparison of HOMO-LUMO gap reduction for different supercell size

We tried to use the same computational settings as before[1]. However, we had to use a different supercell (15×5 instead of 13×13) to fit the molecule inside. Therefore, the k-point density was slightly different (1 k-point per 15 Au atoms instead of 1 k-point per 13 Au atoms), which in principle should not have a big impact on the results as it is below the converged value (1 k-point per 11 Au atoms). To prove the consistency, we performed the same calculations as before for **13ac** in the 15×5 supercell. For the afm state, the HOMO-LUMO gap is reduced and shows only a difference of 0.01 eV (change of 2%), which is within the range of numerical accuracy. This confirms that the supercell has no influence on the HOMO-LUMO gap of the adsorbed acene and allows a comparison between different supercells.

Table S3. HOMO-LUMO gap of gas phase and adsorbed 15ac and 13ac in different electronic states as well as relative difference between them. Energies are given in eV.

Mol	elec. state	HLG(gas)	HLG(ads)	Δ_{rel}(ads-gas)
**15ac**	nm	0.33	0.36	9 %
	afm	0.61	0.49	-19 %
**13ac**[a]	afm	0.57	0.44	-23 %
**13ac**[b]	afm	0.57	0.43	-25 %

[a] 15×5 supercell

[b] 13×13 supercell; values from ref.[1]

8. Gas phase HOMO-LUMO gap of larger acenes

As can be seen in Table S3, the gas phase HOMO-LUMO gap of acenes in the afm state increases from **13ac** (0.57 eV) to **15ac** (0.61 eV). Therefore, the incommensurate oscillation of the HOMO-LUMO gap reported for non-magnetic (closed-shell) DFT calculations[2-4] is still present, although to a lesser extent. For closed-shell DFT computations this is accompanied by a change of symmetry of the HOMO and LUMO close to **10ac**. We attribute this oscillation of the HOMO-LUMO gap to an artificial effect of DFT as mean-field method, even for the afm solution of the spin-polarized variant, since the description lacks static electron correlation. A solution here would be the use of multi-reference methods, for which, in addition, no oscillation of the singlet-triplet gap was observed, in contrast to the DFT results. However, there are two problems with such a multi-reference approach: On the one hand, such methods do not allow for the explicit inclusion of the substrate as the system size would make it unfeasible. On the other hand, such methods no longer possess strictly defined molecular orbitals, which makes the well-established concepts derived from them for the interpretation of STM/STS measurements (e.g. local density of states[5,6]) hard to grasp.

9. Magnetization of adsorbed and gas phase 15ac and adsorbed 13ac

Previously, a decrease of 26% in the magnetization of **13ac** was upon adsorption was computed[1]. For **15ac**, the decrease in magnetization is larger with 36% (Table S4).

In contrast to the previous calculations, the Au(111) slab did not carry a large amount of magnetization (0.086 and 0.073 μ_B, which is < 0.001 μ_B per Au atom). We also did calculations for **13ac** in the 15×5 supercell to compare it to the previous calculations. Surprisingly, the Au slab carried slightly more magnetization than for the **15ac** calculation, but it is still considerably less than before (0.206 vs. 1.973 μ_B, corresponding to < 0.001 vs. 0.003 μ_B per Au atom). However, this has essentially no effect on the magnetization of **13ac** (change of 3%), confirming the conclusion from the previous study that the magnetization of the Au slab has no significant effect on the state of the molecule.

Table S4. Atom-projected magnetization of α and β-spin for 15ac in the gas phase and adsorbed as well as adsorbed 13ac. Magnetization is given in μ_B.

m_{proj}	**15ac**(gas)		**15ac**(ads)		**13ac**(ads)[a]		**13ac**(ads)[b]	
	α	β	α	β	α	β	α	β
C	1.726	1.726	1.102	1.109	1.005	1.040	1.071	1.070
H	0.022	0.022	0.011	0.011	0.011	0.009	0.011	0.011
Au	-	-	0.073	0.086	0.035	0.206	1.973	0.022
sum	1.748	1.748	1.186	1.206	1.051	1.255	3.055	1.103
Δ	0		0.020		0.204		1.952	

[a] 15×5 supercell

[b] 13×13 supercell; values from ref.[1]

10. Magnetization of adsorbed 15ac in the antiferromagnetic state

While Figure 2e in the manuscript shows the average of both adsorbate edges in the gas phase, Figure S5a shows the magnetization distribution of both edges separately (one with α-spin, one with β-spin). It is noticeable that for the adsorbed **15ac** the edge with α-spin has two maxima in the magnetization, while the edge with β-spin has only one maximum. However, this does not appear to be related to the tetraradical character of the gas phase **15ac**, as this has already been observed for **13ac** (Figure S5a)[1]. Instead, it is related to the positions of the carbon atoms above the gold surface atoms. Considering the relative reduction of the individual carbon atoms due to adsorption (Figure S5b), there is always an increased reduction when the carbon atom is on-top of a gold atom (see also Figure S5c). The curves of **13ac** and **15ac** in Fig. S5b are very similar, although it can also be seen there that **15ac** experiences a larger reduction in magnetization than **13ac**.

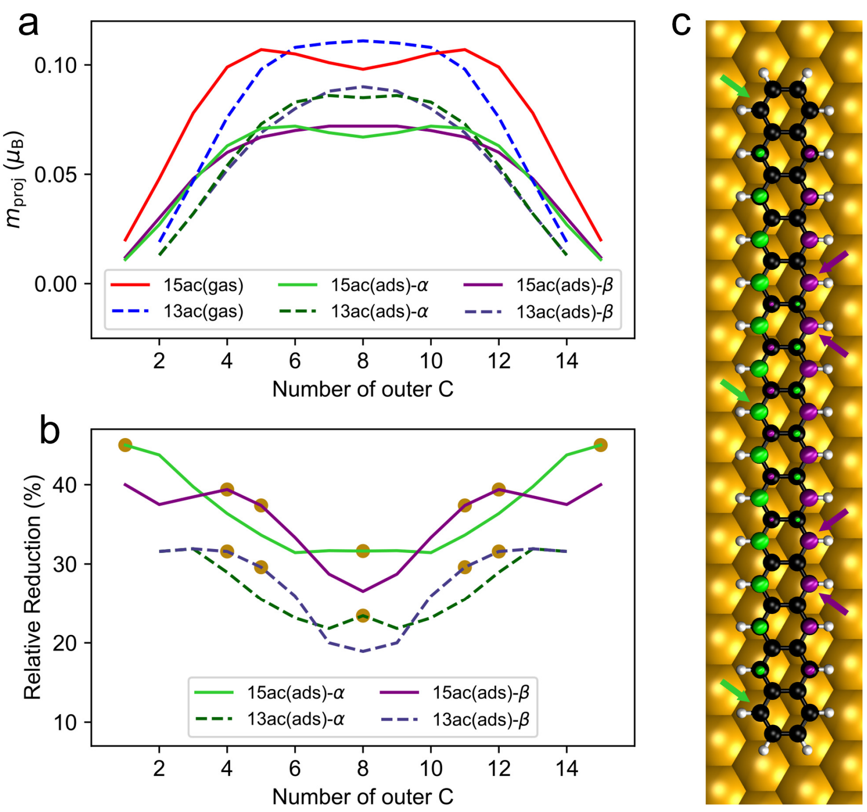

Figure S5. Magnetization of adsorbed 15ac in afm state. In all figures, α-spin is shown in green and β-spin in purple. (a) Atom-projected magnetization for the outer major-spin carbon atoms of gas phase and adsorbed **15ac** and **13ac**, respectively. Values of **13ac** are taken from

ref.[1] and shown for a direct comparison. (b) Relative reduction of the atom-projected magnetization due adsorption for **15ac** and **13ac**, respectively. Values for **13ac** are calculated based on values from ref.[1]. (c) Spin density (ρ_α-ρ_β) of adsorbed **15ac** in afm state. Arrows indicate carbon atoms that are on-top of gold surface atoms.

11. Bias-dependent STM images of the 6Au-15ac complex

The features from the incorporated Au atoms are distinguishable at a wide range of biases, as can be seen in Figure S6 below.

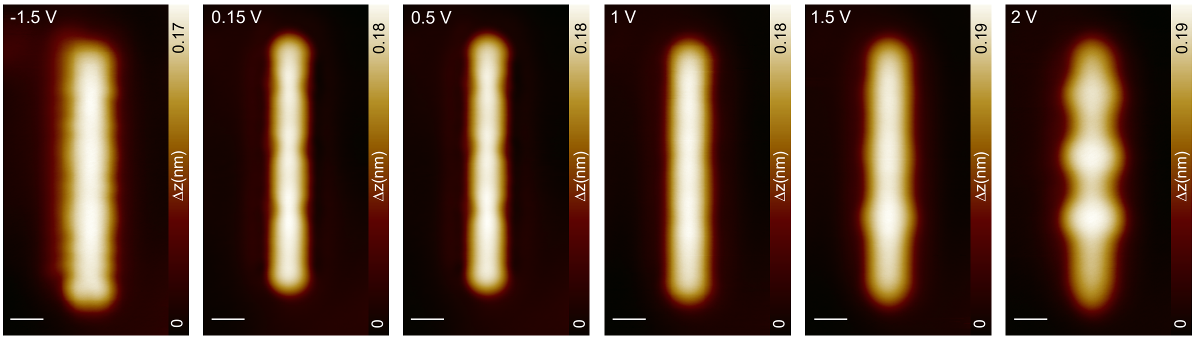

Figure S6. A series of STM images of the 6Au-15ac complex at different bias voltages. A series of STM images of the **6Au-15ac** complex obtained at different bias voltages as indicated in each image. Scale bars: 0.5 nm; Scanning parameters: I_t = 15 pA.

12. Height-dependent nc-AFM images of the 6Au-15ac complex

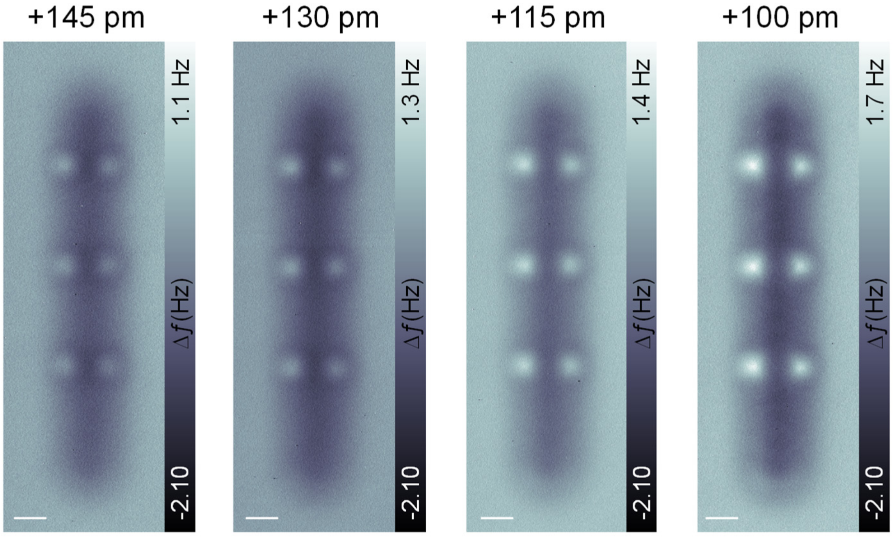

Figure S7. Nc-AFM images of the 6Au-15ac complex. Scale bars: 0.3 nm. Scanning parameters: indicated tip height with respect to open feedback loop at $V_s = 8$ mV, $I_t = 3$ pA.

13. Identification of the gold complex by tip manipulation

The required high sublimation temperature (750 K) leads to cleavages of the three etheno-bridges, and the resulting **15ac** can either react with atomic hydrogen (forming hydroacene) or with gold atoms from the substrate, leading to the pronounced features at the edges. Our STM tip manipulation experiments in Figure S8 shows that the Au atom related features can be reversibly detached and reattached along the edges of the **15ac**, indicating reversible bonding of the adatoms.

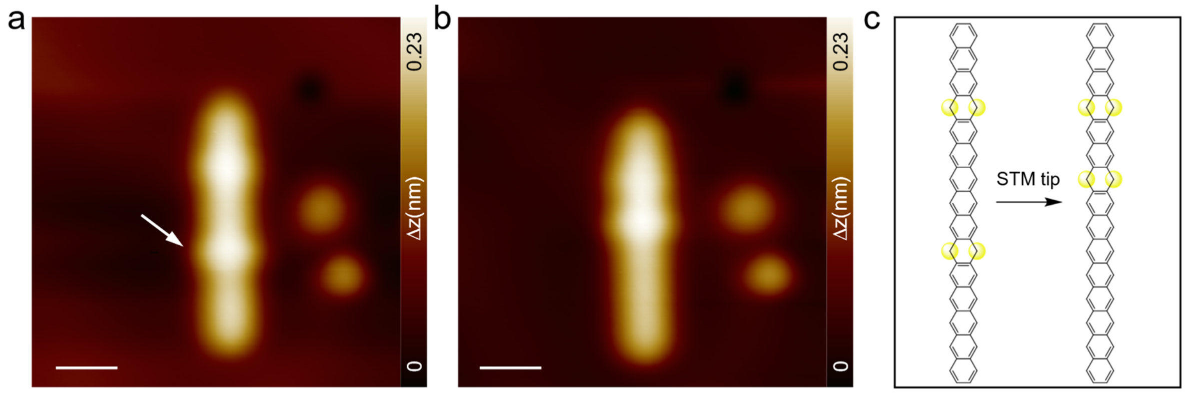

Figure S8. Reversibility of the attachment of Au atoms to the edges of 15ac. (a) **4Au-15ac** adduct showing additional features, and (b) after removing the adatoms at the lower part (white arrow in (a)), the feature from the adatoms now appeared at the position close to the upper part. (c) Chemical models corresponding to the molecules in (a) and (b). Scale bars: 1 nm. Scanning parameters: (a, b) V_s = 2 V, I_t = 15 pA.

Apart from the reversible character of the Au-**15ac** bond, we also directly visualized the complexation of **15ac** with gold. Figure S9 shows a **15ac** molecule generated from its Au complex. As has been observed for other long acenes, the **15ac** molecule is rather mobile even at moderate scanning parameter (Figure S9b). We have tracked its diffusion on surface (Figure S9c-S9f). As can be clearly seen from Figure S9d, additional features attributed to Au atoms appear at the edges after the diffusion step. With further tip manipulation resulting in the removal of the Au atoms, the **15ac** can be regained (Figure S9e and S9f). Considering the cryogenic conditions (4 K) and the UHV (better than 1×10^{-10} mbar) environment, such feature thus can be unambiguously assigned to the gold adatoms. Notably, the removal of a single gold adatom at one edge of the observed adducts also supports that it is not a

hydroacene, as the tip manipulation of a hydroacene in previous work leads to a double cleavage[7,8], i.e., the release of a hydrogen molecule.

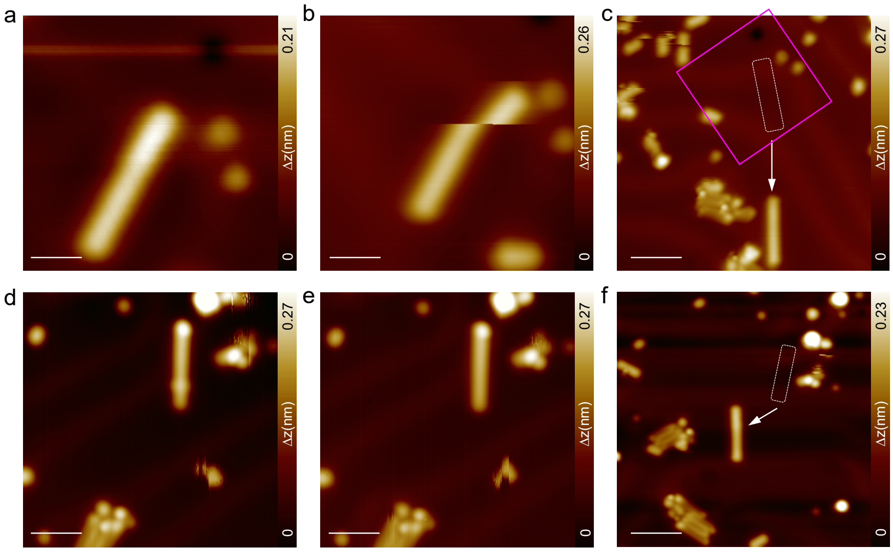

Figure S9. Spontaneous complexation of 15ac with gold atoms. (a) STM of a **15ac** molecule generated by STM tip manipulation. (b) Lateral displacement of the **15ac** at a closer tip-sample separation. (c) The same **15ac** as shown in (a, b) after displacement. The white dashed rectangle marks the original adsorption site of the molecule in (a). (d) The same molecule after further diffusion. Additional features at the lower part of the molecule are now visible. (e, f) The additional features have vanished after STM tip manipulation (e), and a **15ac** molecule can be unambiguously identified again (f). The white dashed rectangle in (f) marks the former adsorption position of the molecule in (d). Scale bars: (a, b) 1 nm; (c) 3 nm; (d, e) 2.5 nm; (f) 6 nm. Scanning parameters: (a) $V_s = 0.5$ V, $I_t = 5$ pA; (b) $V_s = 0.15$ V, $I_t = 35$ pA; (c) $V_s = -0.5$ V, $I_t = 40$ pA; (d-f) $V_s = 2$ V, $I_t = 40$ pA.

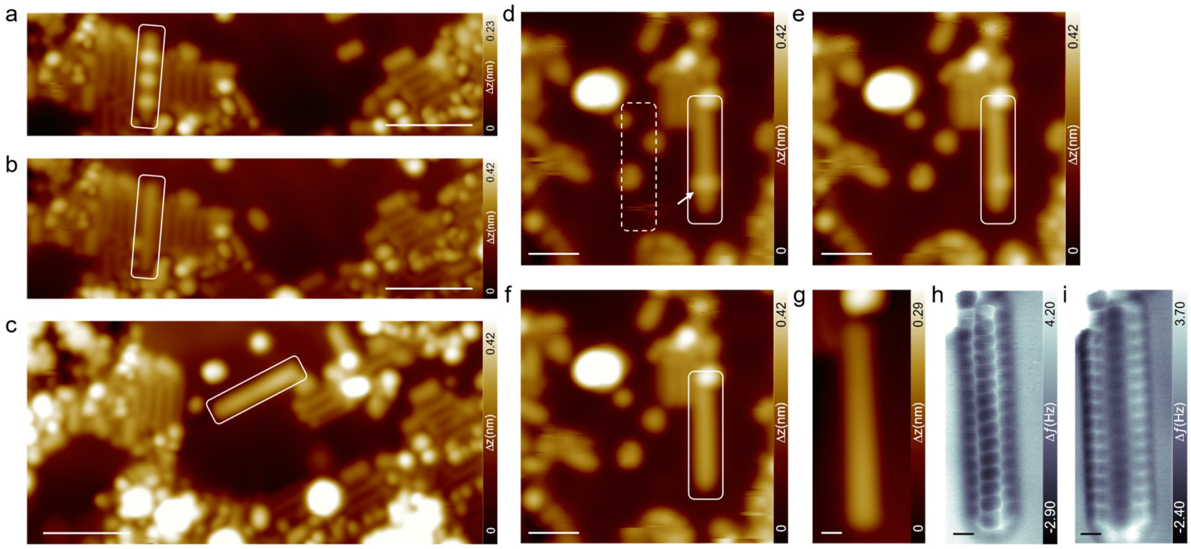

Figure S10. Spontaneous complexation of 15ac with gold atoms. (a) STM image of an intact stretched-out precursor molecule (white dashed square). (b) **15ac** generated from the precursor molecule in (a). (c) The same **15ac** as shown in (b) after cleaning the scanning area with the STM tip. (d) The same molecule as shown in (c) after further stabilization, the white dashed square marks the former adsorption site in (c). Additional features are now visible at the lower part, as indicated by a white arrow. (e) STM image of the same area as in (d). The left adatom is removed by the STM tip. (f) After removing the other adatom at the right edge. (g) STM, (h) CH-nc-AFM and (i) CC-nc-AFM images of the generated **15ac**, as indicated in (f). Scale bars: (a-c) 4 nm; (d-f) 2 nm; (g) 0.4 nm; (h-i) 0.3 nm. Scanning parameters: (a-c) V_s = 0.15 V, I_t = 15 pA; (d-f) V_s = 2 V, I_t = 15 pA; (g) V_s = 0.1 V, I_t = 20 pA.

14. Calculations of Au adatom-15ac complexes

To model the Au adatom bound **15ac**, we tested several possible configurations on the surface of the **6Au-15ac** complex shown in Figure 3a, b. In addition to structures in the 15×5 supercell, these also contained structures in a $(3\sqrt{3}\times9\sqrt{3})$-R30° supercell. The most compelling structure was found in the latter supercell and is shown in Figure S11a. This structure that was the most chemically intuitive (Au atoms are at the right angle to bind to sp^3 hybridized carbon atoms) and fits best with the experimental nc-AFM measurements (Figure 3a and S7), as it has a mirror plane along the long axis and where the upward standing hydrogen atoms are responsible for the bright protrusions. From the final **6Au-15ac** structure, the **5Au-15ac** and **2Au-15ac** complexes were created. These were initially optimized in a non-magnetic state. To our surprise, however, we could not converge to a magnetic state in spin-polarized calculations, neither the doublet for **5Au-15ac**, nor the triplet for **2Au-15ac** (we tested various initializations, structures and number of Au layers). We tried also to enforce a doublet for **5Au-15ac** by fixing the number of spin-up and spin-down electrons. Although the calculation converged, the magnetization of the one electron was delocalized over the whole system and was found more on the surface (60%). Thus, we assume that this is an error of the density functional (PBE) used, which is known to overdelocalize electron densities[9], rather than a physical phenomenon. Due to the large cell size and the large number of gold surface atoms, calculations with higher-order functionals such as hybrid or meta-GGA functionals are not computationally feasible. Therefore, we switched from adsorbed non-magnetic structures to gas phase models, where the respective states could easily be found in single point calculations. Nevertheless, we are convinced that these gas phase models are a good description of the electronic structure of Au adatom complexes. On the one hand, they agree well with the experiment (compare Figure 3e and 3f). On the other hand, we expect the substrate to interact with the molecule only by dispersion interactions, which have no major influence on the electronic structure of the molecule.

To account for structural changes from a non-magnetic to a magnetic structure we made the following approximation: The Au adatoms were frozen at the positions of the optimized adsorbed non-magnetic structure. Additionally, we froze the z-coordinates of the 4 outermost

carbon atoms (2 at each end of **15ac**). In this way, the molecule stays flat as if there is a surface underneath (otherwise, the ends of **15ac** bend upwards due to the sp^3-hybridization of the carbon atoms to which the Au adatoms bind).

For the **2Au-15ac** complex, we could find three electronic states: a closed-shell singlet (CS), an open-shell singlet (OS), and a triplet (T). Both singlet states are very close in energy, with the OS being 0.2 kJ/mol more stable than the CS (Figure S11b). The triplet state is higher in energy than both singlet states with 17 kJ/mol (Figure S11c), which confirms the experimentally found absence of a Kondo resonance.

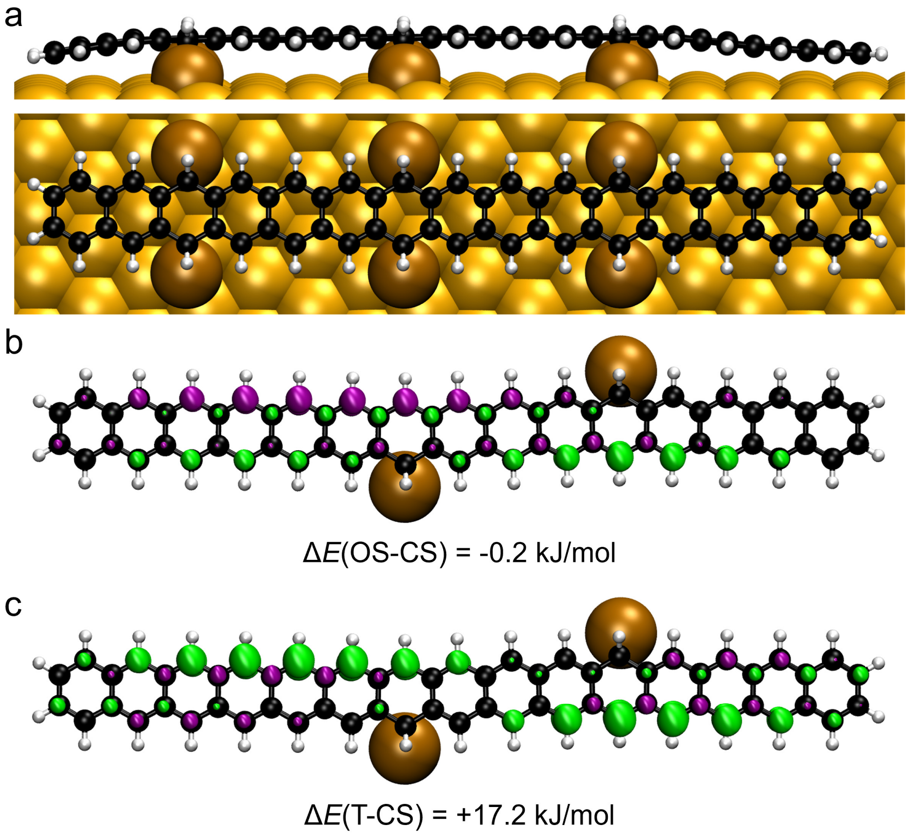

Figure S11. Theoretical modeling of Au adatom-15ac complex. In (b) and (c), α-spin is shown in green and β-spin in purple. (a) Side and top view of adsorbed **6Au-15ac** structure. (b) Spin density (ρ_α-ρ_β) of gas phase model structure of the **2Au-15ac** complex in open-shell singlet (OS) state with energy relative to closed-shell singlet (CS) state given below. (c) Spin density of gas phase model structure of the **2Au-15ac** complex in triplet (T) state with energy relative to closed-shell singlet (CS) state given below.

15. STS measurements of an odd-numbered and an even-numbered gold complex

The low-energy bias STS measurements of the **5Au-15ac** complex show a variation in the Kondo peak intensity (Figure S12a and S12b), which is due to the varying spatial distribution of the spin density. The dI/dV curves (Figure S12d) for the **2Au-15ac** complex in Figure S12c is featureless, suggesting a (closed-shell or open-shell) singlet electronic state.

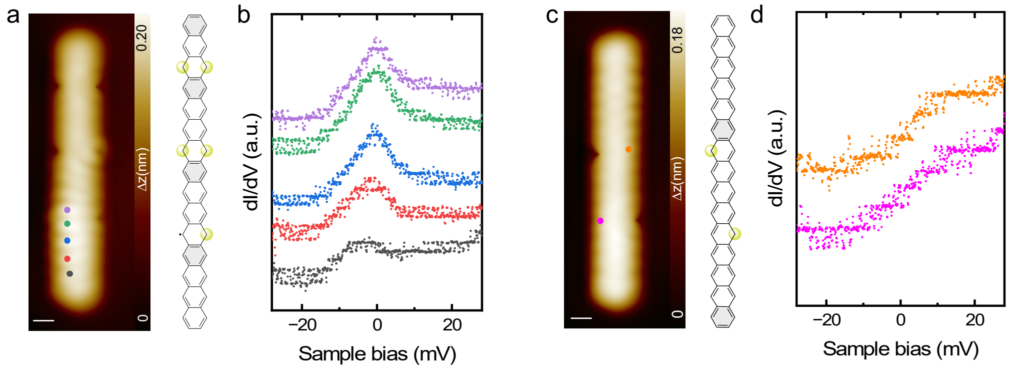

Figure S12. Low-energy d*I*/d*V* curves acquired on the Au-15ac complex. (a) STM image of the **5Au-15ac** complex as shown in Figure 3 in the main text and its corresponding chemical structure (right). (b) d*I*/d*V* curves taken at positions denoted by dots with the same colors in (a). (c) The **2Au-15ac** complex as shown in Figure 3 in the main text and its corresponding chemical structure (right). (d) d*I*/d*V* curves taken at positions denoted by dots with the same colors in (c). Scale bars: (a, c) 0.3 nm. Scanning parameters: (a, c) V_s = 10 mV, I_t = 5 pA.

16. Overview STM images of the 6Au-15ac complex before and after tip manipulation

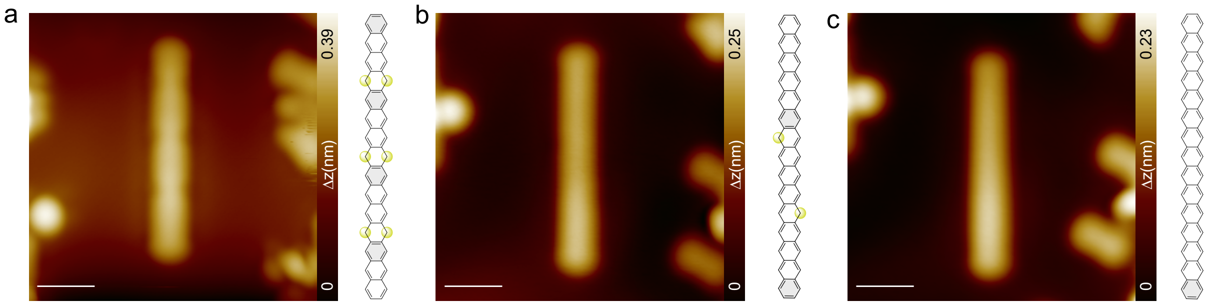

Figure S13. STM images of the 6Au-15ac complex and its further transformation. (a-c) STM image and chemical structure of the **6Au-15ac** complex before and after tip manipulation. Scale bars: (a-c) 1 nm. Scanning parameters: (a) V_s = 0.15 V, I_t = 8 pA; (b) V_s = 0.12 V, I_t = 7 pA; (f) V_s = 0.15 V, I_t = 10 pA.

17. Manipulation and characterization of a 6Au-15ac complex

As shown below in Figure S14a, the Au-related indentations of this **6Au-15ac** complex have slightly different contrast in STM (compare e.g. bottom right and top right indentations). This is attributed to different adsorption sites of the individual Au atoms, possibly resulting from a different adsorption site of the whole complex, compared to the complex shown in Figure 3a. Using the STM tip, we are able to also generate the complexes with odd numbers of gold adatoms. Figure S14b (**5Au-15ac**) and Figure S14c (**3Au-15ac**) show pronounced features originating from Kondo scattering at the sites where a gold atom was removed (white arrow). Remarkably, such a feature is also preserved for the complex with only one gold adatom (Figure S14d), for which a Kondo peak can be detected (Figure S14e), suggesting a doublet state. This observation agrees well with the spin excitation feature observed for **15ac**, thus convincingly confirming the open-shell singlet ground state of **15ac**.

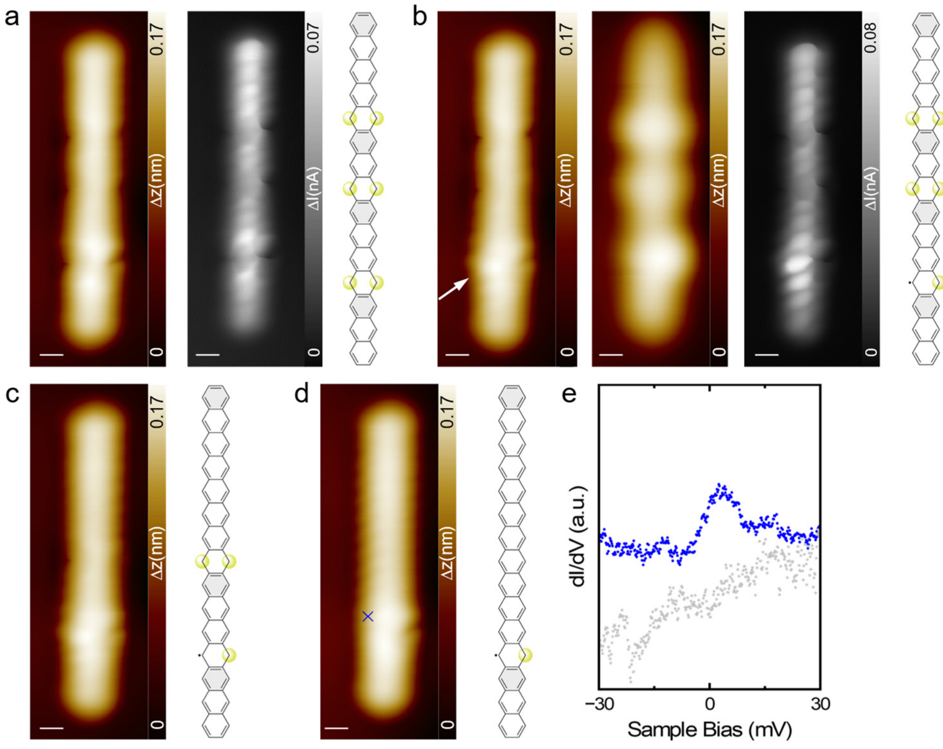

Figure S14.Tip-induced structural transformation of a 6Au-15ac complex. (a) (left to right) STM, BR-STM and the corresponding chemical structure of a **6Au-15ac** complex. (b) STM images with different tunneling parameters (see below), BR-STM and the chemical structure of the **5Au-15ac** complex. (c) STM image and chemical structure of the **3Au-15ac**

complex. (d) STM image and chemical structure of the **1Au-15ac** complex. (e) dI/dV curve measured at the spot indicated by a blue cross in (d). Scale bar: 0.3 nm. Scanning parameters: STM: (a, c, d) V_s = 0.15 V, I_t = 20 pA; (b) V_s = 0.15 V, I_t = 20 pA (left) and V_s = 2 V, I_t = 10 pA (right).

18. Manipulation and characterization of a 5Au-15ac complex

For complexes with odd numbers of gold adatoms, the Kondo resonance can be unambiguously identified from STS measurements, as has been extensively discussed above (for an additional example, see Figure S15a-S15c). It is further confirmed by removing the adatom close to the radical site in an odd-numbered Au complex, as can be seen from Figure S15d. The odd-numbered Au complexes (n_{Au} = 5 in Figure S15a and n_{Au} = 3 in S15c) show pronounced features at the bottom left of the molecules due to Kondo scattering. After manipulation, these features have vanished in the resulting even-numbered Au complexes (n_{Au} = 4 and n_{Au} = 2, respectively). The structure of the **15ac** obtained by removing all the adatoms are confirmed by nc-AFM measurement (Figure S15e, right).

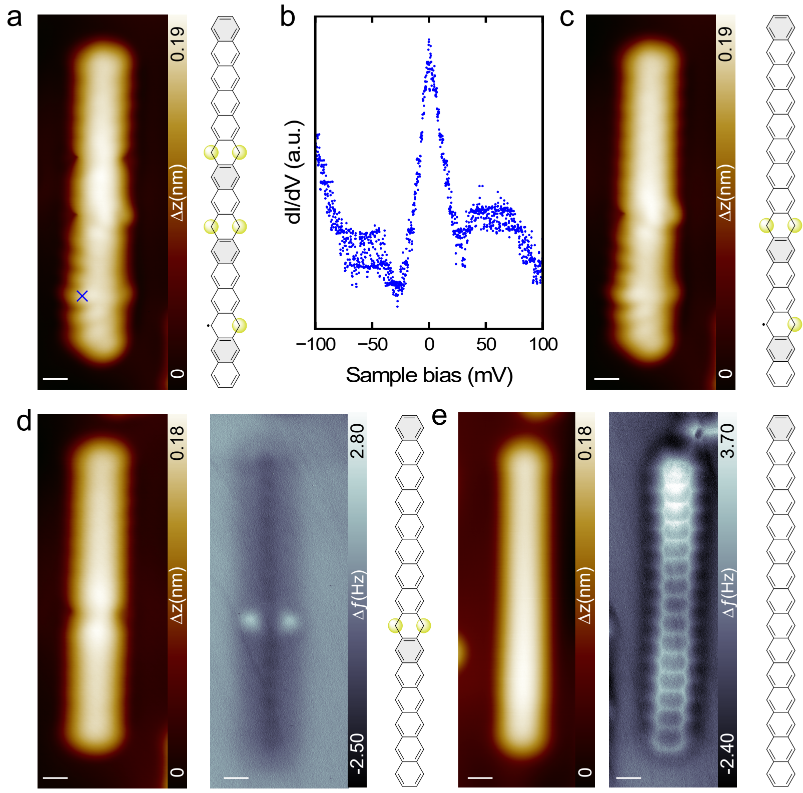

Figure S15. Tip-induced structural transformation of a 5Au-15ac complex. (a) STM image and the corresponding chemical structure of a **5Au-15ac** complex after removing one gold adatom from a **6Au-15ac** complex. (b) dI/dV curved acquired at the spot marked by a cross in (a). (c) STM image and the chemical structure of the **3Au-15ac** complex. (d) STM (left) and nc-AFM image (middle) and the corresponding chemical structure of a **2Au-15ac**

complex. (e) STM and nc-AFM image of the same molecule as in (a-d), after removing all the gold adatoms. Scale bar: 0.3 nm. Scanning parameters: STM: (a, b, d) V_s = 0.15 V, I_t = 15 pA; (e) V_s = 0.15 V, I_t = 20 pA.

19. NMR and mass spectra

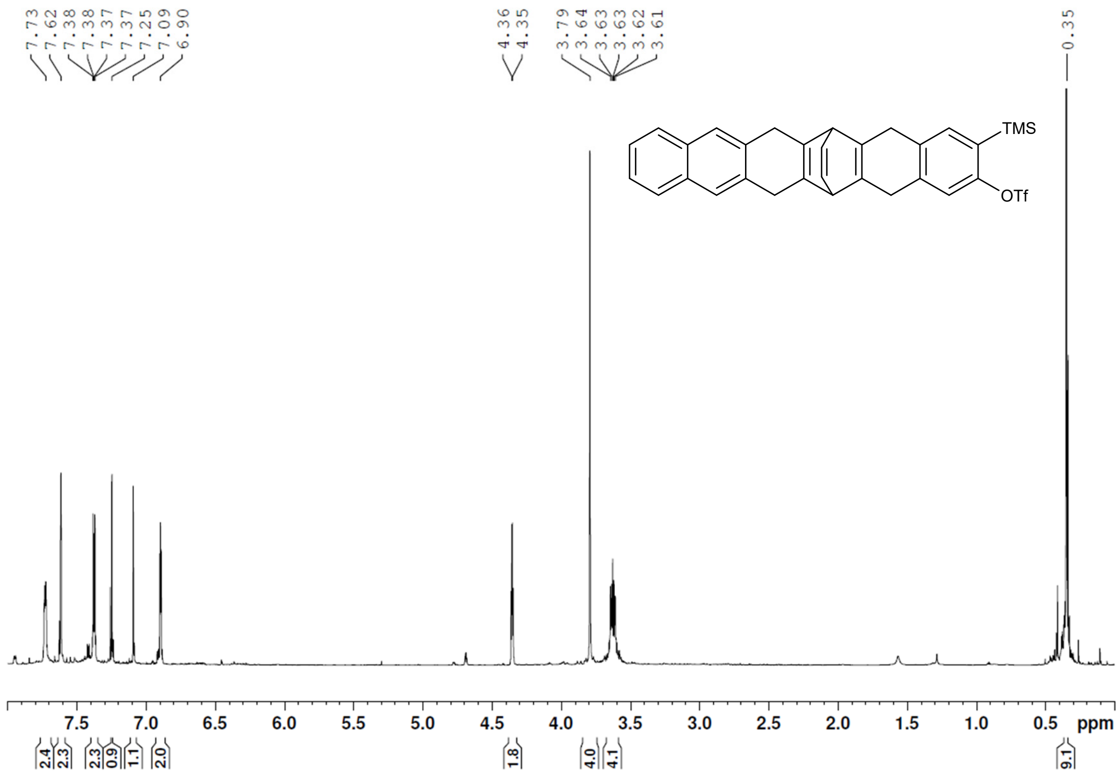

Figure S16. ^{1}H NMR spectrum (700 MHz) of 4 in $CDCl_3$.

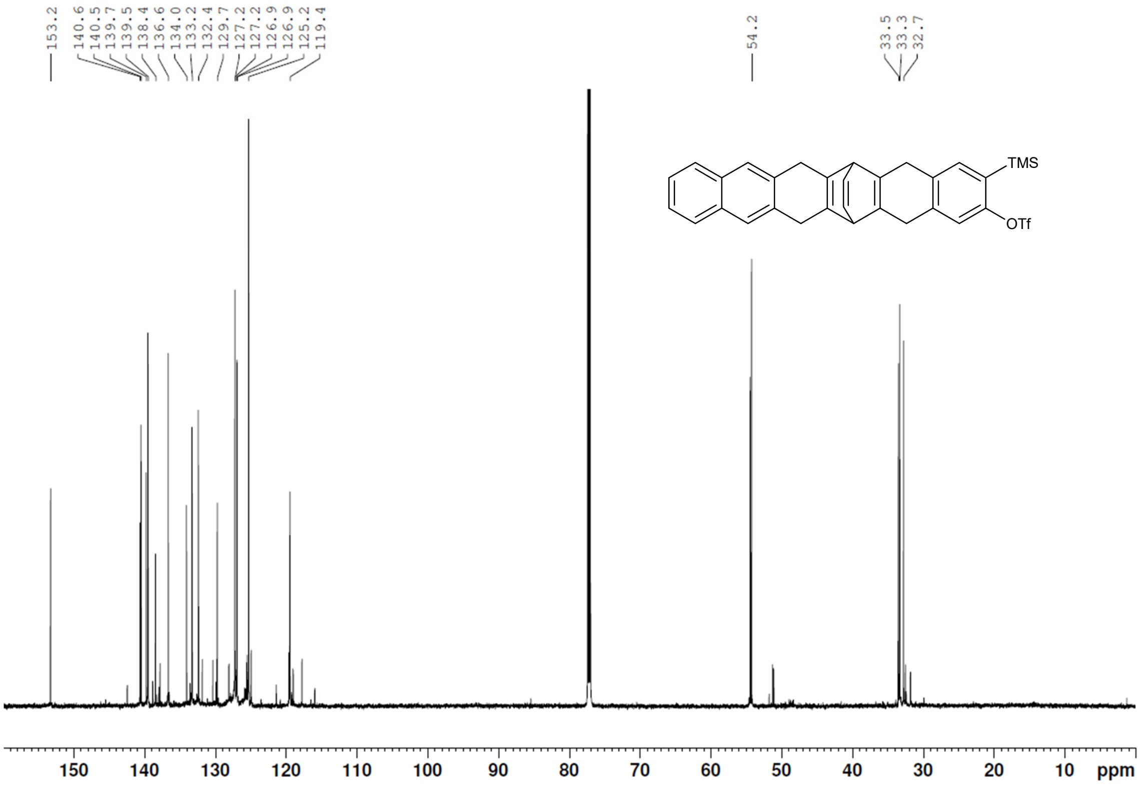

Figure S17. ^{13}**C NMR spectrum of 4 (176 MHz, $CDCl_3$).**

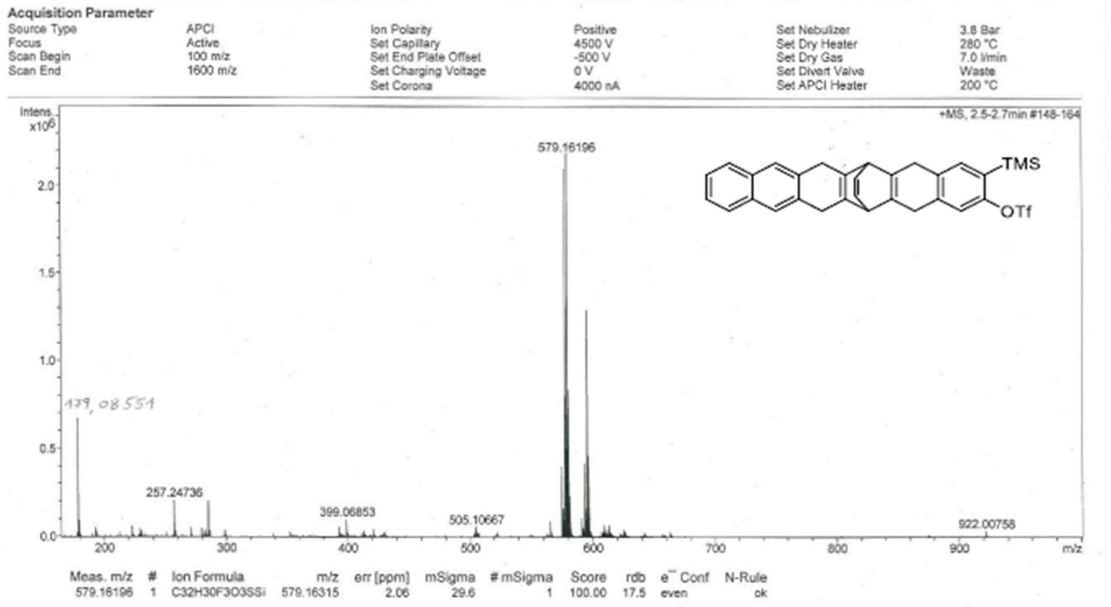

Figure S18. APCI mass spectrum (high resolution, full spectrum) of 4.

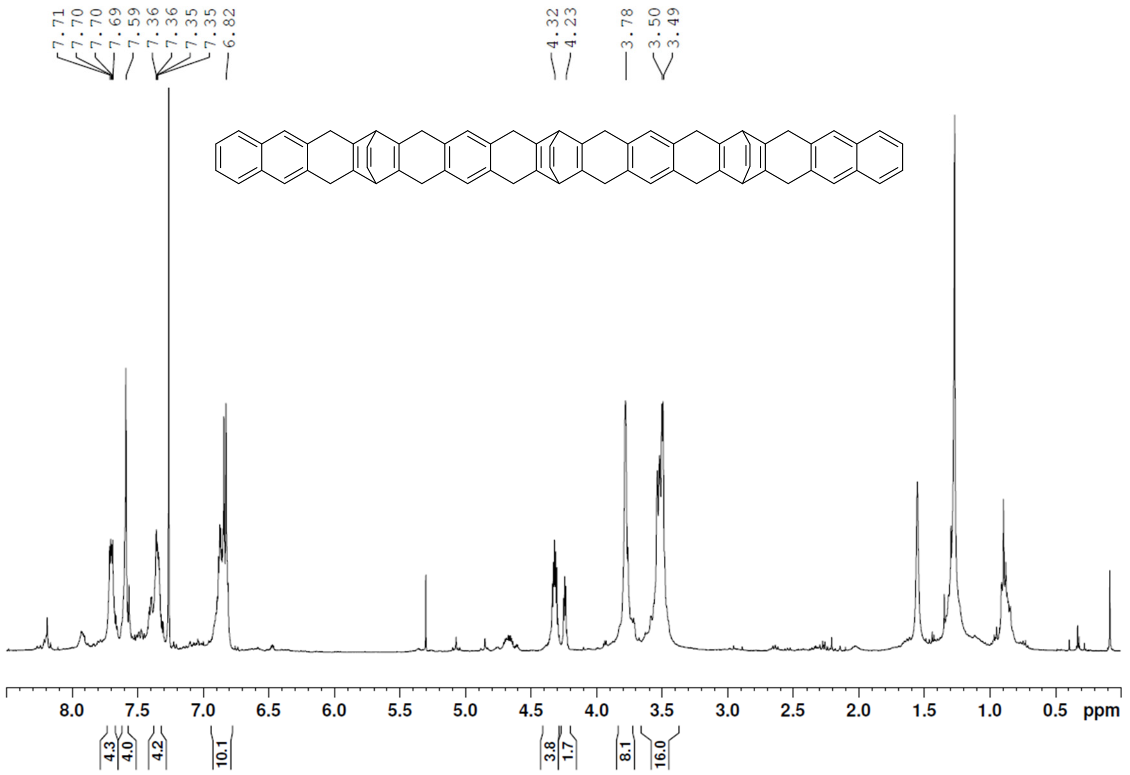

Figure S19. ^{1}H NMR spectrum (400 MHz) of 6 in $CDCl_3$.

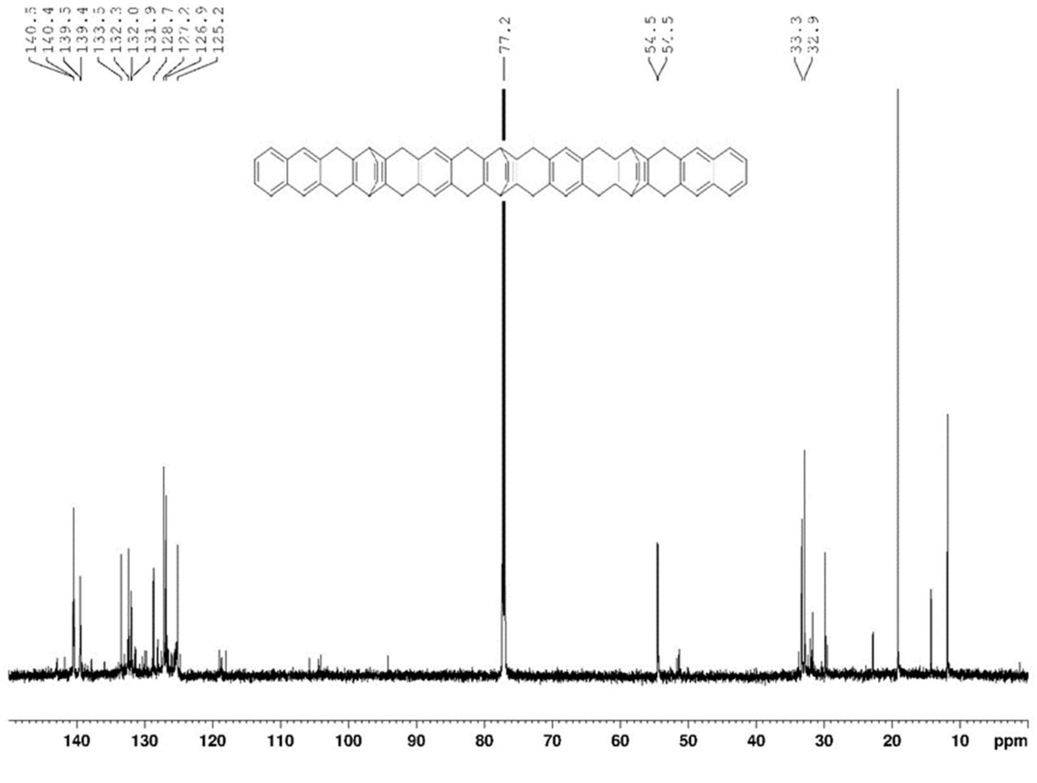

Figure S20. ^{13}C NMR spectrum of 6 (176 MHz, $CDCl_3$).

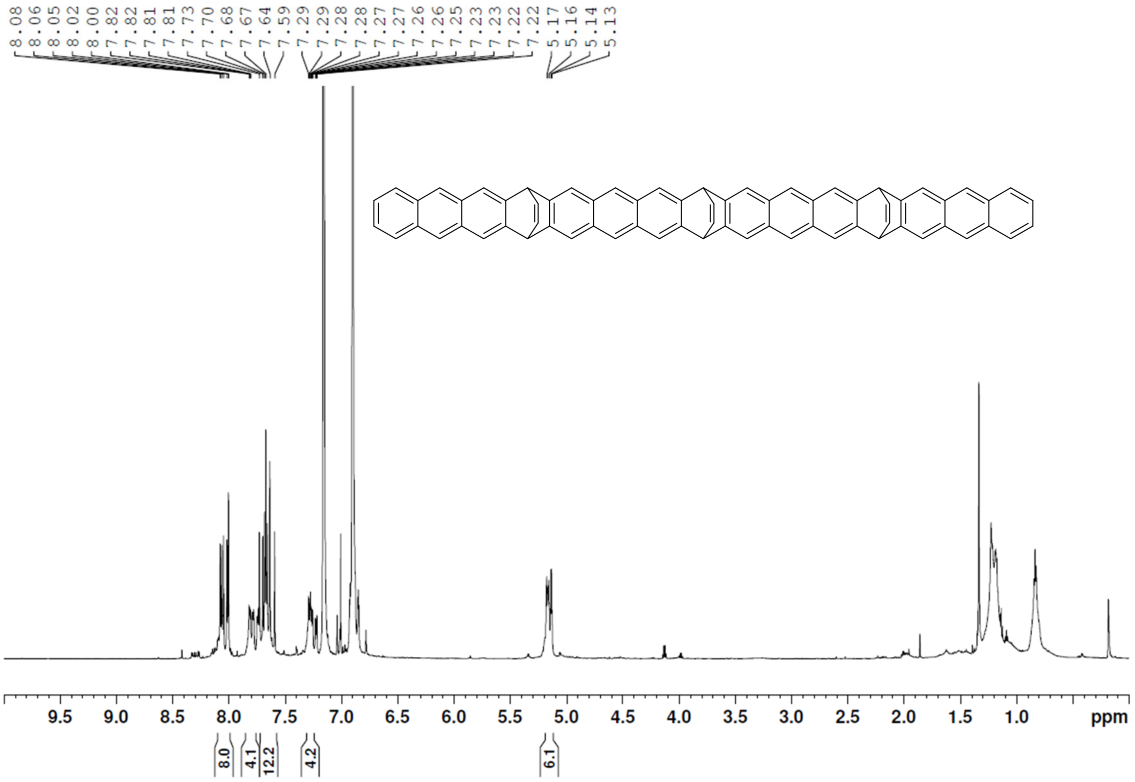

Figure S21. **^{1}H NMR spectrum (700 MHz) of 1 in $C_6D_4Cl_2$.**

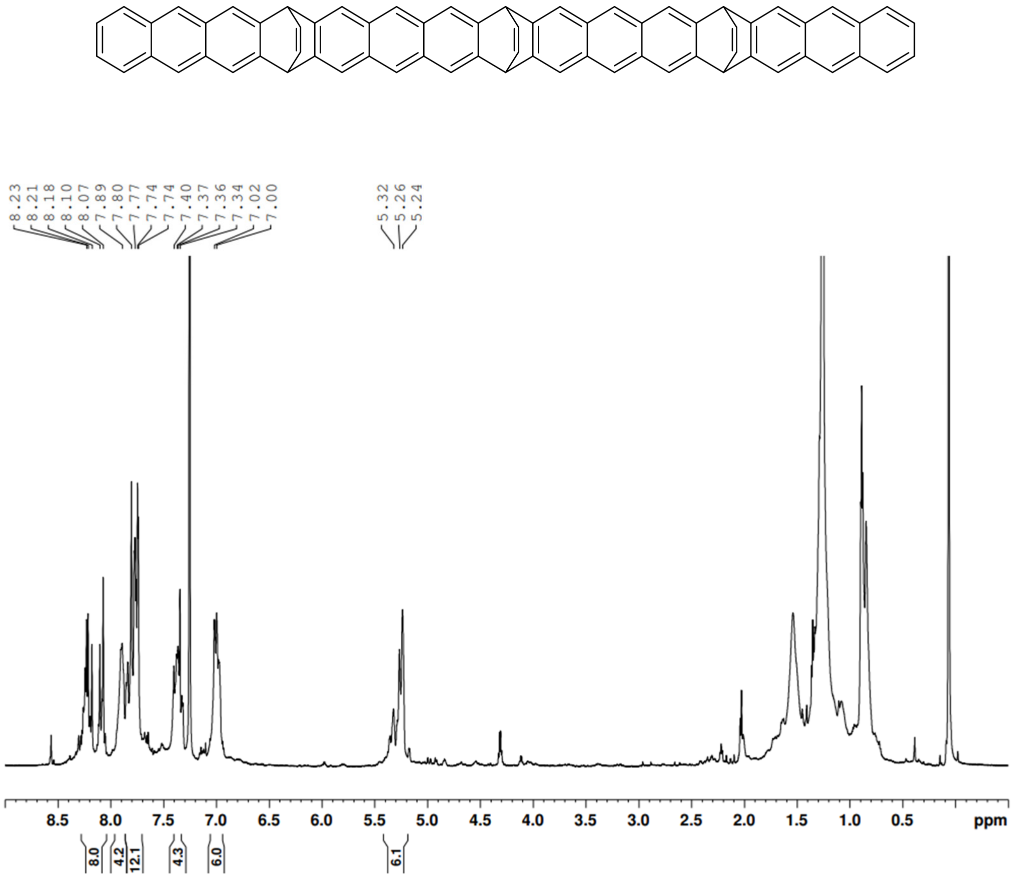

Figure S22. **^{1}H NMR spectrum of 1 (700 MHz, CS_2 : $CDCl_3$, 10 : 1).**

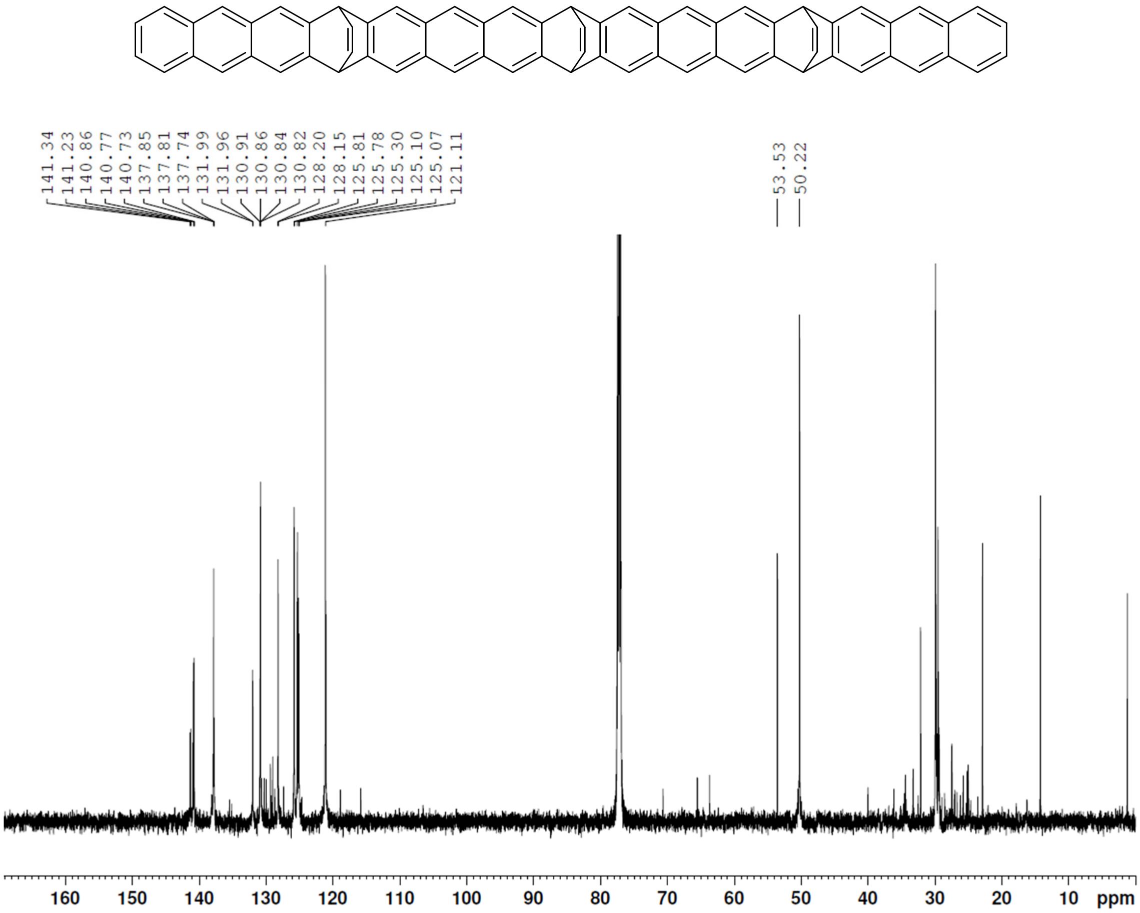

Figure S23. ^{13}C **NMR spectrum of 1 (323 K, 151 MHz,** $CDCl_3$**).**

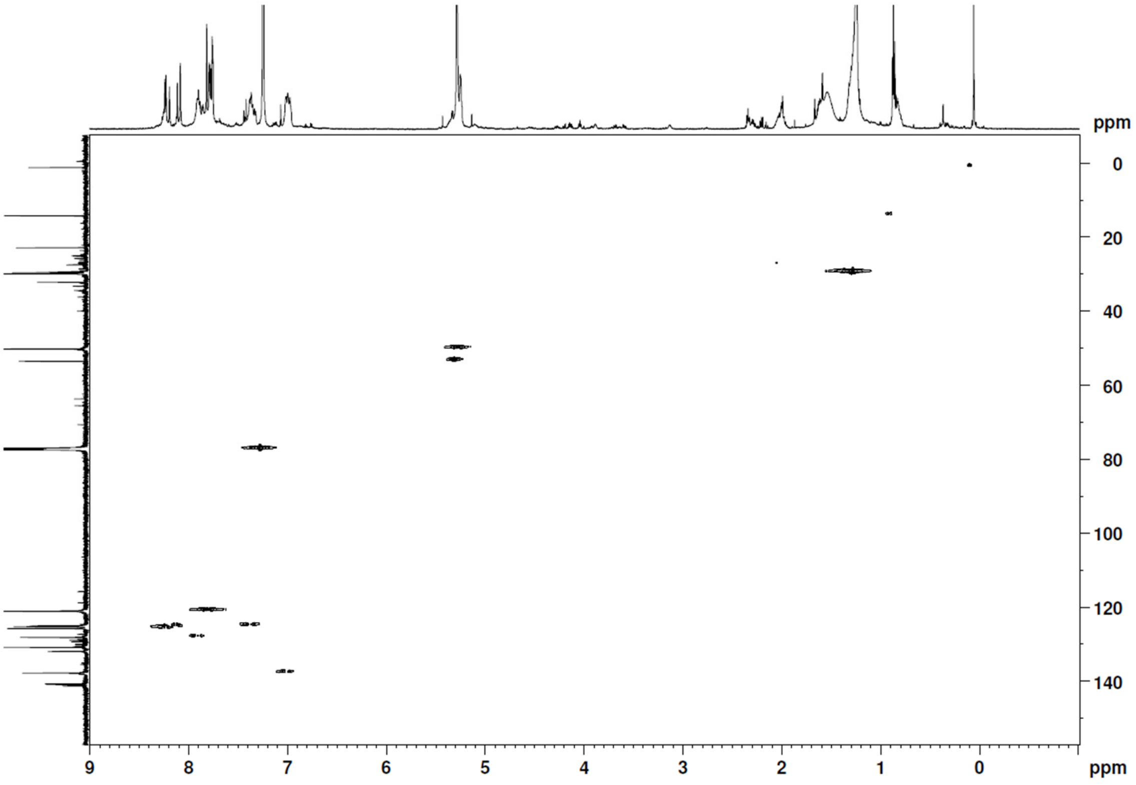

Figure S24. HSQC NMR spectrum of 1 in $CDCl_3$ with 600 MHz (^{1}H) and 151 MHz (^{13}C).

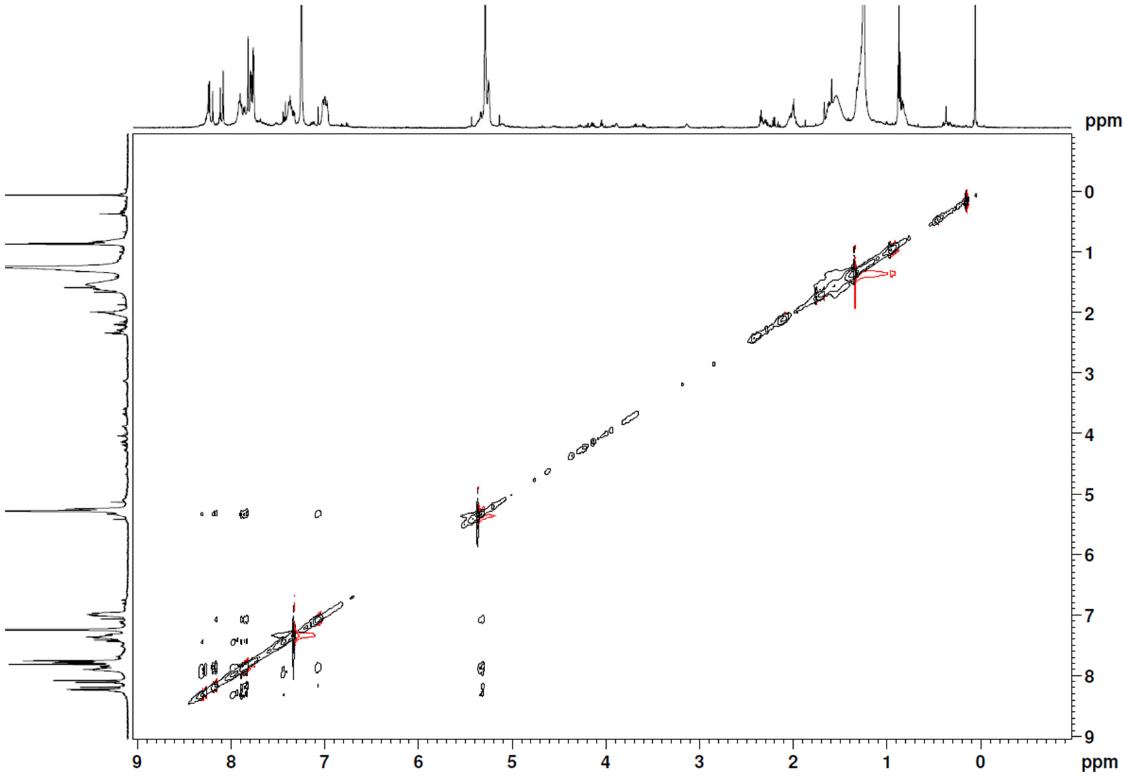

Figure S25. NOESY NMR spectrum of 1 (600 MHz) in $CDCl_3$.

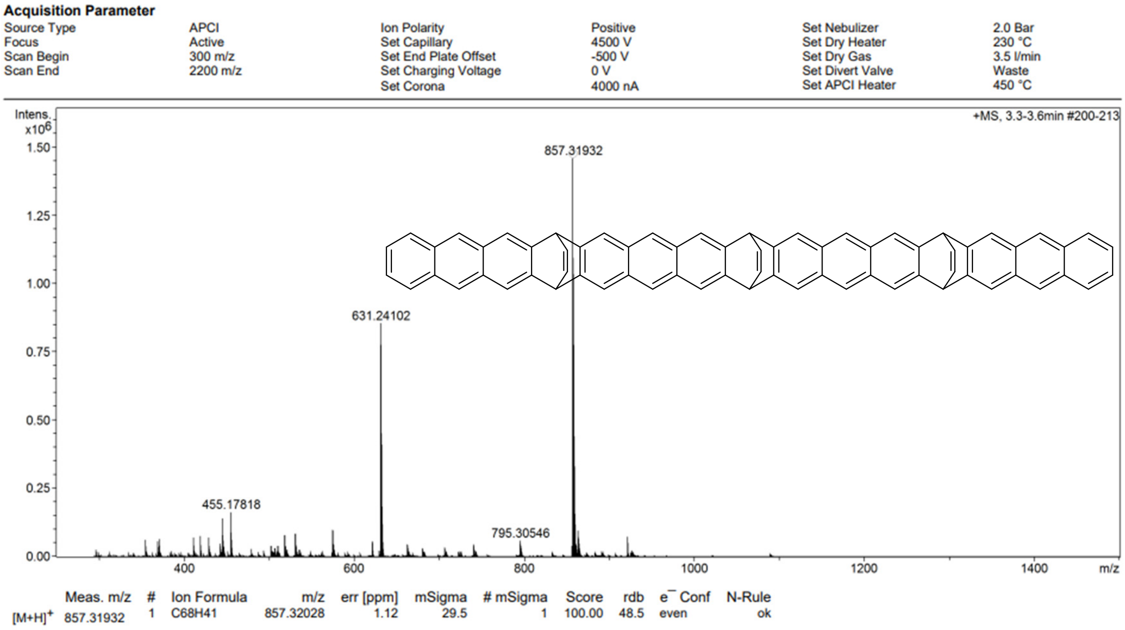

Figure S26. APCI mass spectrum (high resolution, full spectrum) of 1.

References

1. Ruan, Z.; Schramm, J.; Bauer, J. B.; Naumann, T.; Bettinger, H. F.; Tonner-Zech, R.; Gottfried, J. M. Synthesis of Tridecacene by Multistep Single-Molecule Manipulation. *J. Am. Chem. Soc.* **2024**, *146*, 3700-3709. DOI: 10.1021/jacs.3c09392.

2. Korytár, R.; Xenioti, D.; Schmitteckert, P.; Alouani, M.; Evers, F. Signature of the Dirac cone in the properties of linear oligoacenes. *Nat. Commun.* **2014**, *5*, 5000. DOI: 10.1038/ncomms6000.

3. Schmitteckert, P.; Thomale, R.; Korytár, R.; Evers, F. Incommensurate quantum-size oscillations in acene-based molecular wires—Effects of quantum fluctuations. *J. Chem. Phys* **2017**, *146*. DOI: 10.1063/1.4975319.

4. van Setten, M. J.; Xenioti, D.; Alouani, M.; Evers, F.; Korytár, R. Incommensurate Quantum Size Oscillations of Oligoacene Wires Adsorbed on Au(111). *J. Phys. Chem. C* **2019**, *123*, 8902-8907. DOI: 10.1021/acs.jpcc.8b12213.

5. Tersoff, J.; Hamann, D. R. Theory of the scanning tunneling microscope. *Phys. Rev. B* **1985**, *31*, 805-813. DOI: 10.1103/PhysRevB.31.805.

6. Selloni, A.; Carnevali, P.; Tosatti, E.; Chen, C. D. Voltage-dependent scanning-tunneling microscopy of a crystal surface: Graphite. *Phys. Rev. B* **1985**, *31*, 2602-2605. DOI: 10.1103/PhysRevB.31.2602.

7. Zuzak, R.; Dorel, R.; Krawiec, M.; Such, B.; Kolmer, M.; Szymonski, M.; Echavarren, A. M.; Godlewski, S. Nonacene Generated by On-Surface Dehydrogenation. *ACS Nano* **2017**, *11*, 9321-9329. DOI: 10.1021/acsnano.7b04728.

8. Zuzak, R.; Dorel, R.; Kolmer, M.; Szymonski, M.; Godlewski, S.; Echavarren, A. M. Higher Acenes by On-Surface Dehydrogenation: From Heptacene to Undecacene. *Angew. Chem. Int. Ed.* **2018**, *57*, 10500-10505. DOI: 10.1002/anie.201802040.

9. Cohen, A. J.; Mori-Sánchez, P.; Yang, W. Challenges for Density Functional Theory. *Chem. Rev.* **2012**, *112*, 289-320. DOI: 10.1021/cr200107z.
